# Platonic Micelles: Monodisperse Micelles with Discrete Aggregation Numbers Corresponding to Regular Polyhedra

**DOI:** 10.1038/srep44494

**Published:** 2017-03-14

**Authors:** Shota Fujii, Shimpei Yamada, Sakiko Matsumoto, Genki Kubo, Kenta Yoshida, Eri Tabata, Rika Miyake, Yusuke Sanada, Isamu Akiba, Tadashi Okobira, Naoto Yagi, Efstratios Mylonas, Noboru Ohta, Hiroshi Sekiguchi, Kazuo Sakurai

**Affiliations:** 1 Department of Chemistry and Biochemistry, University of Kitakyushu, 1-1 Hibikino, Wakamatsu-ku, Kitakyushu, Fukuoka 808-0135, Japan; 2 Department of Chemical Science and Engineering, Ariake National College of Technology, 150 Higashihagio-Machi, Omuta, Fukuoka 836-8585, Japan; 3 Japan Synchrotron Radiation Research Institute (JASRI/SPring-8), 1-1-1 Kouto, Sayo, Sayo, Hyogo 679-5198, Japan

## Abstract

The concept of micelles was first proposed in 1913 by McBain and has rationalized numerous experimental results of the self-aggregation of surfactants. It is generally agreed that the aggregation number (*N*_*agg*_) for spherical micelles has no exact value and a certain distribution. However, our studies of calix[4]arene surfactants showed that they were monodisperse with a defined *N*_*agg*_ whose values are chosen from 6, 8, 12, 20, and 32. Interestingly, some of these numbers coincide with the face numbers of Platonic solids, thus we named them “Platonic micelles”. The preferred *N*_*agg*_ values were explained in relation to the mathematical Tammes problem: how to obtain the best coverage of a sphere surface with multiple identical circles. The coverage ratio *D(N*) can be calculated and produces maxima at *N* = 6, 12, 20, and 32, coinciding with the observed *N*_*agg*_ values. We presume that this “Platonic nature” may hold for any spherical micelles when *N*_*agg*_ is sufficiently small.

Surfactants and lipids comprise of hydrophilic polar head groups and hydrophobic tails, commonly long hydrocarbon chains. When these molecules are dispersed in aqueous solutions, the hydrophobic tails tend to avoid unfavorable interactions with polar water, while the head groups favorably interact with water. This consequently leads to the formation of aggregates with a certain size, called “micelles”. The term of micelle was first used by J. W. McBain in 1913 to describe an aggregate of soap molecules and the first model of the spherical micelles (Supplementary Information Figure S1), which is still widely used today, was proposed by G. S. Hartley^1^. There are numerous practical applications of micelles such as soaps, detergents, and drug delivery carriers and their chemistry and physics are still a major subject in modern science.

As shown in Hartley’s model, the polar headgroups usually form the exterior of the aggregate and the hydrophobic moieties form the interior. Their volumetric balance is the main determinant of the micelle shape. Israelachvili^2^ introduced a thermodynamic parameter (*a*_e_) related to the equilibrium area per molecule at the aggregate interface and proposed a geometrical criterion that needs to be fulfilled for the micelles to take a spherical shape;


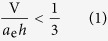


where *V* and *h* are the volume and length of the surfactant tails, respectively^2^. The factor of 1/3 is related to the geometrical relations for the volume and surface area of a sphere made up of *N* surfactants, subject to the constraint that the radius of the sphere *R* cannot exceed the extended tail length *h*. We can imagine that a particular number of cones can assemble to form a spherical shape. This concept is called packing parameter principle and has been widely used because it provides an intuitive insight on the aggregation phenomena of micelles. By the same geometrical analogy, the aggregation number of spherical micelles (*N*_*agg*_) can be expressed by;


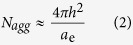


Tanford was the first to formulate the thermodynamics of the micelle formation (S11)^3^. His theory and its improvements^4^,^5^, and additionally recent computer simulations^6^,^7^ based on these theories are quite consistent with various experimental results for wide range of materials^8^. It is generally accepted that *N*_*agg*_ gradually changes with the surfactant concentration and other solvent conditions such as the ionic strength and *N*_*agg*_ has a certain distribution. However, we recently observed an interesting phenomenon that seemingly cannot be rationalized within the above framework. In this paper, we overview our experimental results and propose a possible theoretical explanation for this finding.

## Materials and Methods

**Figure 1:**
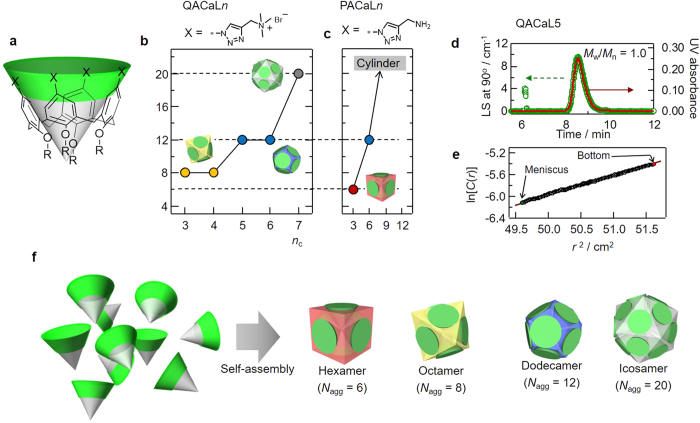
Alkyl chain length dependence of the aggregation number (*N*_*agg*_) of two calix[4]arene derivatives; QACaLn (quaternary amine) and PACaLn (primary amine). (**a**) Schematic illustration of the chemical structure of the calix[4]arene derivatives and their conical shape, where green and white parts represent hydrophobic and hydrophilic regions, respectively, and X and R denotes hydrophilic functional groups and alkyl chains, respectively. (**b**,**c**) *N*_*agg*_ plotted against the number of carbon atoms in one tail (*n*_*C*_). (**d**,**e**) Experimental evidences illustrating monodispersity (upper : aFFF coupled with MALS and UV, and lower: AUC). (**f**) A schematic illustration of calix[4]arene derivatives arranged on the faces of the Platonic solids.

We have newly synthesized 18 calix[4]arene-based surfactants as presented in Fig. 1a, whose chemical structures and codes are shown in Table S1. These molecules consist of four headgroups and four alkyl tails, and presumably take a conical shape as presented in Fig. 1a. As a surfactant other than the calix[4]arene ones, we used a bacterial lipopeptide: surfactin, which consists of a heptapeptide unit (Glu-Leu-D-LeulVal-Asp-D-Leu-Leu) and a beta-hydroxy fatty acid, obtained from Kaneka Corporation (SF-S04), Japan. Synchrotron small angle X-ray scattering (SAXS) was carried out at BL40 at SPring 8, Japan to determine the molecule shape as well as *N*_*agg*_. Analytical ultracentrifugation (AUC) and asymmetric flow field-flow fractionation (aFFF) coupled with multi-angle static light scattering (SLS) were used to determined *N*_*agg*_ and dispersity (see the Supplementary Information). The critical micellar concentrations (CMC) were determined and listed in Table S1, which were in the range 0.4–100 μM. *N*_*agg*_ did not change when the concentration was less than approximately 50–100 × CMC.

## Results and Discussion

### Finding monodisperse micelles with aggregation numbers coinciding with the face numbers of Platonic solids

A series of calix[4]arene surfactants designated as QACaL*n* (which has four quaternary amino headgroups and *n* is the number carbon atoms of one tail, as shown in Fig. 1b) were examined by use of SAXS, SLS, and AUC (see S3). It was clear that these molecules form spherical micelles in water. We plotted*N*_*agg*_ against the number of carbons in the alkyl chain (*n*_*C*_) (Fig. 1b). As *n*_*C*_ increases from 3 to 7, *N*_*agg*_ increases discretely from 8 to 12, and then to 20. An increase in *N*_*agg*_ is expected from the packing parameter theory, which predicts that *N*_*agg*_ ∝ (*n*_*C*_)^2^. However, we would have expected a continuous rather than a discrete increment. Figure 1d shows the SLS (green) and UV (red) signals of an aFFF fractogram (see S4) for QACaL5. The micelles show a peak at 8.5 min, and the LS and UV signals completely overlap. The SLS intensity is proportional to the sample concentration and molecular weight, whereas the UV absorbance depends only on the concentration. Therefore, the overlap of these signals indicates that the molecular weight is constant over the entire peak. The dispersity index, defined as *M*_w_/*M*_n_, is determined to be 1.0, for this sample, where *M*_n_ and *M*_w_ are the number and weight averaged molecular weights, respectively. The monodispersity of the sample was confirmed with AUC as *M*_z_/*M*_w_ = 1.0, also indicated by the linearity of the ln *C(r) vs. r*^2^ plot (Fig. 1e), where *M*_z_ is the z-averaged molecule weight, and *r* and *C(r*) are the distance from the rotating center and the concentration at *r* after reached equilibrium. When *n*_*C*_ ≥ 8, the dispersity index becomes larger than 1.0 and the *N*_*agg*_ distribution widens. As reported for PACaL*n*, which has a primary amine headgroup^9^, we observed a similar discrete increment of *N*_*agg*_ and monodispersity (Fig. 1c). Here, as *n*_*C*_ increases from 3 to 6, *N*_*agg*_ increases from 6 to 12.

We have synthesized several other calix[4]arene derivatives with sugar (GalCaL3), amino acid (CCaL3 and ECaL3)^10^, amine dendrimer (G1CaL3), choline phosphate (CPCaL3 and CPCaL6)^11^, and poly(ethylene oxide)(PEGnCaL5) headgroups as summarized in Table S1. Some examples exhibit similar monodispersity and discreteness of *N*_*agg*_. So far, such monodispersed aggregation numbers that we have encountered are 6, 8, 12, 20, and 32. It is quite surprising that 6, 8, 12, and 20 coincide with the face numbers of Platonic solids, as illustrated in Fig. 1f. Here, Platonic solids are regular, convex polyhedra and only five polyhedra meet these criteria.

### Discrete changes in *N*_*agg*_ with changing solvent conditions

**Figure 2:**
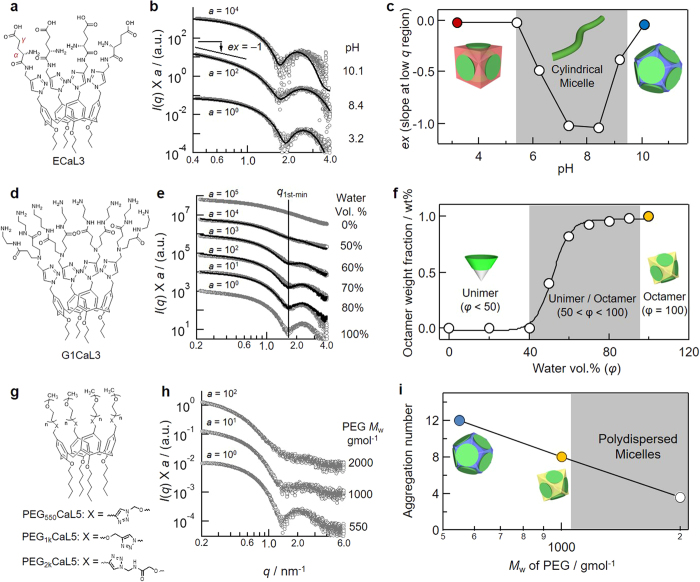
Discrete change of the aggregation number (*N*_*agg*_), when solvent conditions are continuously changed (upper two systems) and when the volume of non-ionic headgroup is changed (bottom). (**a**,**d**,**g**) Chemical structures of the calix[4]arene derivatives. (**b**,**e**,**h**) SAXS profiles for each sample, where the profiles are vertically shifted for ease of comparison. (**c**) *N*_*agg*_ = 6 and 12 are appeared, reflecting the charged positions (i.e., *h* in Eq. 2) of the glutamic acid head at acid and base. (**f**) Equilibrium between *N*_*agg*_ = 8 and *N*_*agg*_ = 1 describes the SAXS profiles. (**g**–**i**) The bottom panels show that non-ionic amphiphilic calix[4]arene derivatives (g) bearing PEG also form platonic micelles: PEG chain length dependence of SAXS in (**h**) and of *N*_*agg*_ in (i).

We have examined how the discreteness and monodispersity can be maintained when solvent conditions are continuously changed (Fig. 2). Figure 2b shows the pH dependence of SAXS for ECaL3 (glutamic acid head, Fig. 2a) and the exponent *ex* in *I(q*) ∝ *q*^*ex*^ at low *q* is plotted against pH in Fig. 2c, where *I(q*) and *q* are the scattering intensity and magnitude of the scattering vector, respectively. The exponent *ex* at low *q* is related to the geometrical dimension of scattering objects; *ex* = 0, −1, and −2 indicate finite sizes for all dimensions, rod-like objects, and disk-like objects, respectively^12^. As shown in Fig. 2b,c, *ex* = *0* was observed at pH < 5.4 and pH = 10. This indicates that ECaL3 forms micelles of a finite size, and fitting the SAXS data confirmed the formation of spherical micelles in these pH ranges (see S5). In intermediate pH values, *ex* decreases, reaching *ex* = −1 at 7 < pH < 8.5. Intermediate values of *ex* between −1 and 0 indicate the cylinder is not long enough to gives *ex* = −1, meaning short cylindrical micelles with various lengths. Aggregation number analysis with SAXS and SLS (see S5) indicated that v *N*_*agg*_ = 6 and = 12 at pH < 5.4 and pH = 10, respectively. Furthermore, we confirmed the monodispersity at both conditions. Spherical micelle formation at both acidic and basic conditions and its transition to cylinders can be explained as follows: ECaL3 has four glutamic acids as headgroups which contain both a primary amine and a carboxyl group; i.e., they have an amphoteric nature. Therefore, the headgroups can be charged and thus create repulsive interactions at both acidic and basic conditions, leading to larger *a*_*e*_ in these conditions than in neutral ones. The larger *a*_*e*_ values favor the formation of spheres and the smaller ones the formation of cylinders, according to Eq. 1, which can explain the sphere to cylinder transition upon pH change. At low pH when the amino groups are charged, the distance from the hydrophobic core surface at which the charge is located is smaller compared to when the carboxyl groups are charged at high pH. Therefore, the electrostatic repulsions will be smaller for the carboxyl case at high pH compared to the amino case at low pH. The result would be a larger aggregation number for the carboxyl case at high pH compared to the amino case at low pH.

Figure 2e shows the SAXS profiles of G1CaL3 (amido-amine head, Fig. 2d) in water/methanol solvents. As the water volume percent (φ) increases, the intensity minimum of the scattering profiles deepens. It should be noted that the position of the minimum 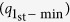 does not change. This behavior contrasts strongly with that of other micelle systems (see S6), where 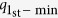 shifts continuously towards smaller *q* with increasing water content, because of the continuous increase in the micellar size. Knowing that G1CaL3 forms monodisperse octamers in water and remains a unimer in methanol, the unchanged 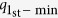 means that the water/methanol solutions contain only unimers and octamers and the smeared scattering profiles at lower φ is owing to the increase of the unimer content. Based on this assumption, the scattering intensity profile [*I(q, φ*)] at an intermediate *φ* can be expressed as a linear combination of *I(q, φ* = 0) and *I(q, φ* = 1), after multiplying by an appropriate coefficient. The solid lines fitted to the experimental data points are calculated values, showing an excellent agreement, thus supporting our assumption. From the coefficient used, we can estimate the weight fraction ratio of the octamer to the unimer (*w.f*.) (see S7) and the obtained *w.f*. is plotted against *φ* in Fig. 2f. The increase of water content sigmoidally changes *w.f*. from 0 to 1 around *φ* = 50 According to the conventional micellization theory, the distribution of *N*_*agg*_ becomes broader near the CMC due to larger thermal fluctuations^2^, which is seemingly not the case in the present system.

### Other systems to show the monodispersity and discreteness of *N*_*agg*_

Electrostatic repulsions are not necessary for the creation of discreteness and monodispersity. When we attached long polyethylene glycol chains to calix[4]arene (PEG_550_CaL5 and PEG_1k_CaL5), they formed dodecamers and octamers, in accordance with the volume of the PEG head group as shown in Fig. 2g–i. The monodispersity of this system is confirmed with aFFF (S8). One may also suggest that these features are characteristically observed for the above mentioned calix[4]arene systems because of the rigidity and trapezoid-like body of the calix[4]arene building block. However, there have been a few reports in the literature showing similar discreteness and monodispersity for micelles made from surfactants of different building blocks. Khelfallah *et al*.^13^ prepared a water-soluble amphiphilic cylindrical brush-coil block copolymer. They observed that their polymer formed micelles consisting of 4 or 5 block copolymers on average and the size distribution was quite narrow. Böttcher *et al*.^14^,^15^ synthesized an amphiphilic fullerene derivative and the molecules showed monodispersity comparable to our systems, and they named their systems shape persistence micelles. They observed their micelles with cryo-electron transmission microscopy and reconstructed the 3D-image. Although electron microscopy does not provide absolute molecular weight, their tomographic image suggested that six molecules of the fullerene derivative make up one micelle.

**Figure 3:**
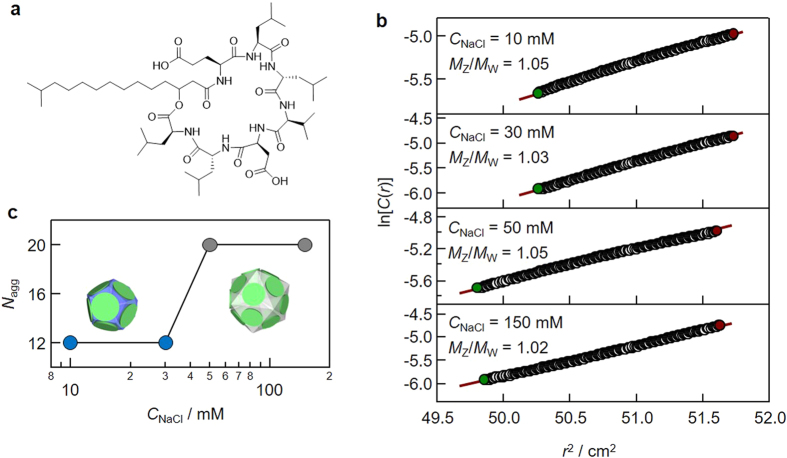
Self-assembly of surfactin. (**a**) Chemical structure, (**b**) plots of ln *C(r) vs. r*^2^ for AUC, where the linearity indicates monodispersity and the meniscus position indicates the magnitude of the molecular weight. The position was discretely changed with an increase of NaCl concentration (*C*_NaCl_). (**c**) *N*_*agg*_ plotted against *C*_NaCl_.

Surfactin is a bacterial lipopeptide containing large negatively charged cyclic peptides with two carboxyl groups originating from aspartate and glutamate (Fig. 3a)^16^. When we determined *N*_*agg*_ as a function of *C*_NaCl_ with AUC (Fig. 3b,c), *N*_*agg*_ changed from 12 and 20 as *C*_NaCl_ increased. The similar salt concentration dependence of *N*_*agg*_ was observed by another group with small angle neutron scattering^17^. We confirmed that no peptide conformational change was induced by NaCl using circular dichroism (S9). The SAXS profiles showed a sharp intensity minimum, similar to the calix[4]arene derivatives (S9) and the monodispersity was confirmed with AUC (Fig. 3b). The present surfactin results further confirm that calix[4]arene derivatives are not only the case to show monodispersity with a *N*_*agg*_ chosen from Platonic solid face numbers. It would have been better to demonstrate the existence of “Platonic micelles” with conventional surfactants such as SDS and DTAC. However, these surfactants have rather large *N*_*agg*_ making it experimentally difficult to determine *N*_*agg*_ with accuracy better than ±1 owing to an inevitable error of at least 5%. In another word, for example, we may not be able to distinguish 48 and 49, (although we will discuss later, where *N*_*agg*_ = 48 may be a possible configuration of the platonic micelles, but *N*_*agg*_ = 49 is not).

### Proposal of Platonic micelles and their relationship to the mathematical problem of the best coverage of a spherical surface with multiple circles

**Figure 4:**
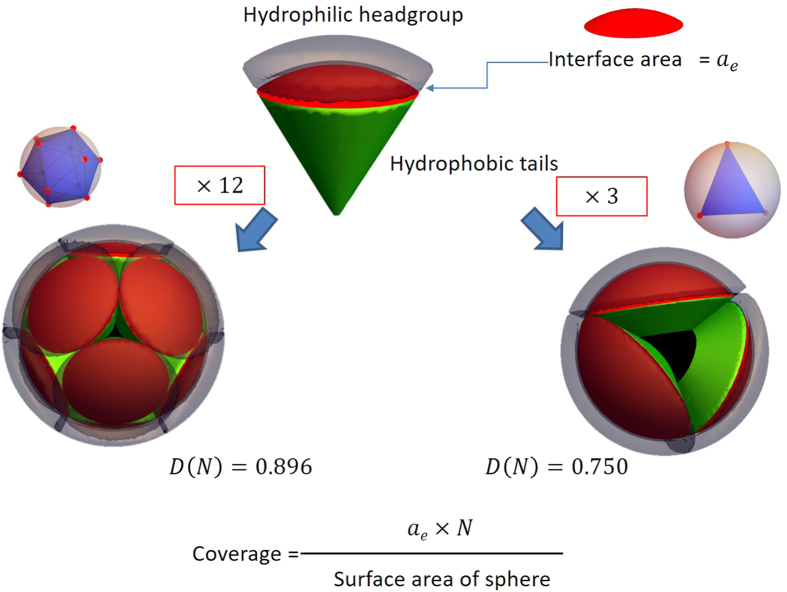
Two examples of the best packing of multiple circles on a sphere surface (*N* = 3 and 12). The Tammes problem and its analogy to how multiple cone shaped surfactants form spherical micelles to cover their hydrophobic tail domain (green) with the interface area (red) acting as the spherical caps. For *N* = 3, even with the best configuration, the hydrophobic domain is significantly exposed and the coverage *D(N*), defined by the ratio of the total area of the caps to the surface area of the sphere, is 0.75. For *N* = 12, the hydrophobic domain is mostly covered by the interfacial area and *D(N*) = 0.896.

**Figure 5:**
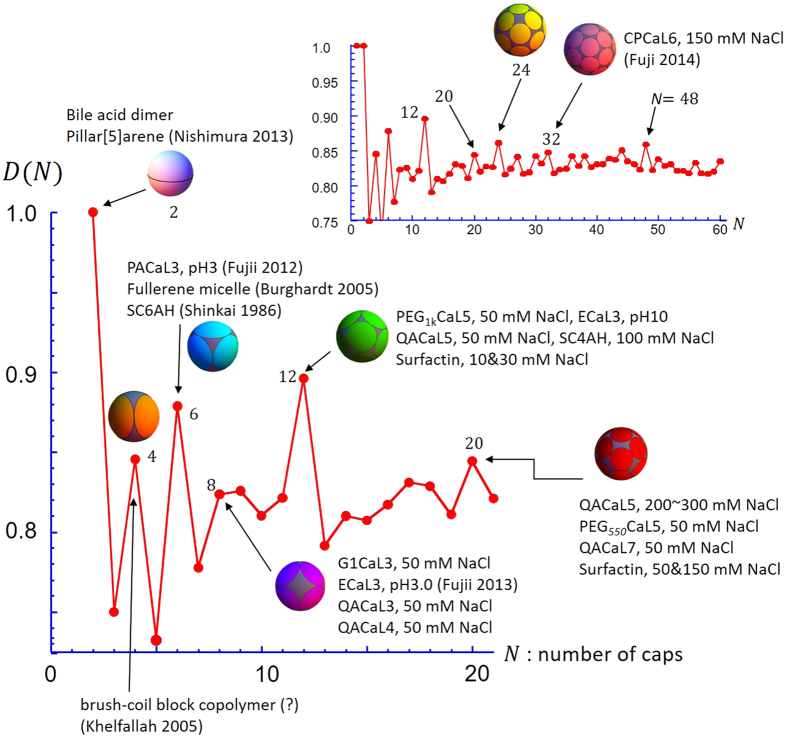
The coverage *D(N*) of the Tammes problem plotted against the number of caps *N* and the monodisperse aggregation numbers observed so far as well as reported in the literatures^9^,^10^,^11^,^13^,^14^,^23^,^25^ are indicated. The colored circles covering the grey spheres show the geometry of the best configuration for the respective *N*.

There have been no reports for such discreteness and monodispersity in conventional micelles. Here, it should be noted that the classical micellar thermodynamics shows that the dispersity in *N*_*agg*_ can become quite narrow when the hydrophobic interfacial energy is large enough^18^. In this case, it can be also shown that *N*_*agg*_ becomes almost independent of surfactant concentration. Therefore, the discreteness in *N*_*agg*_ is the most intriguing and peculiar feature of the present system. Hereinafter, we will focus on this discreteness. Generally, normal micelles have larger *N*_*agg*_ than the present systems. Smaller *N*_*agg*_ mean larger *a*_*e*_ according to Eq. 2. If *a*_*e*_ is large and *N*_*agg*_ is small, the way in which the hydrophobic tails are covered to achieve the minimum free energy may differ from the systems with a large *N*_*agg*_. This issue may be related to the mathematical problem of how to efficiently cover the surface of a sphere with multiple spherical caps of equal radius. In geometry this is called the “best packing on a sphere” or Tammes problem^19^,^20^,^21^ (see S10). For example, as illustrated in Fig. 4 (right hand), we can have the maximum coverage with three spherical caps when the center of each cap is located on the vertices of an equilateral triangle touching the sphere. In this case, the coverage density *D(N*) is 0.75. For 12 caps (left), we can have a larger coverage: *D(N*) = 0.879 and the centers of the caps have the same configuration as the vertices of a regular icosahedron. Here, *D(N*) is defined as the ratio of the sum of the cap areas to that of the sphere surface, as illustrated in the bottom of Fig. 4. It should be noted that *D(N*) is not a simple monotonic function of *N*. Figure 5 plots *D(N*) against the number of caps *N*. For large *N, D(N*) is almost constant at 0.82–0.83. On the other hand, for small *N*, larger *D(N*) values are obtained at certain numbers such as *N* = 6, 12, and 20. It is surprising that these numbers coincide with our observed values of *N*_*agg*_. On analogy, it can be reasonably assumed that there are certain numbers of molecules that can more efficiently cover spherical micelles than other numbers (i.e., more decrease in the interfacial free energy originated from unfavorable contact water and the hydrophobic tails). One may think that real molecules do not behave like rigid spherical caps, but the hard-core potential (i.e., Tammes problem) is not a necessary condition for an oscillating *D(N*) pattern. A similar simulation with the Coulomb potential exhibits local maximums in the same *N*s with the Tammes problem (S10). Therefore we can presume that *N*_*agg*_ = 6, 12, and 20 are more likely to be encountered in real micelles. The monodispersity can be explained in the same framework. When a certain *N*_*agg*_ is strongly favorable, most aggregates will consist of this number of molecules and the system becomes monodisperse.

Figure 5 summarizes all the monodisperse systems that we observed so far as well as some examples from the literatures. CPCaL6, which has zwitterion choline phosphate headgroups (Table S1), has *N*_*agg*_ = 32. It is notable that there is a local maximum of *D(N*) at *N* = 32 and its configuration is the same as carbon-60. *N* = 2 gives *D(N*) = 1.0 because two hemispheres can completely cover a sphere. The molecular configuration cannot be identical to hemispheres, but we have two examples for *N*_*agg*_ = 2. One is a dimer of bile acids^22^, and the other is a pillar[5]arene based amphiphilic molecule^23^. We have several examples at *N*_*agg*_ = 8, but the coverage *D(N*) at *N* = 8 is not so large. Although it is much larger than 7, it is slightly smaller than 9. The *N* = 8 configuration is not a regular hexahedron but an augmented triangular prism (i.e, a skewed cube). The symmetry of this configuration is still quite high and this may explain why we encounter *N*_*agg*_ = 8. By the way, it is not clear at the moment how large *N*_*agg*_ must be to consider *D(N*_*agg*_) to be constant. This may depend on the chemical structure. Here, we would like to point out that there are maxima of *D(N*) at *N* = 32 and 48 and these numbers are normally observed *N*_*agg*_ for conventional surfactants such as alkyl sulfates.

### Molecular dynamics simulations

For PACaL3 (Fig. 1c, *N*_*agg*_ = 6), we constructed a rigid body model of the hexameric micelle with the program SASREF^24^ which shows good agreement with the experimental SAXS data (Fig. S12-1). Using this model as a guide, we designed a starting model for molecular dynamics (MD) simulations. SAXS profile was calculated from each MD model and the similarity Jaccard index (*J*_ind._) between the experimental and calculated profiles was also determined. While the total energy oscillated around a constant value (Fig. S12-2), *J*_ind._fluctuated considerably. Probably, our MD simulations overestimate the thermal fluctuations of the atomic positions.

**Figure 6:**
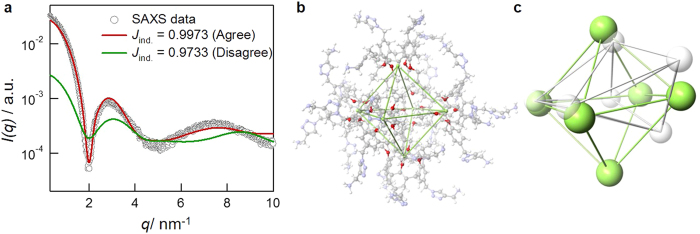
Results of MD. (**a**) Comparison of the experimental SAXS profiles and those calculated from the MD models. (**b**) An atomic representation of the model with the maximum *J*_ind._ with the positions of the oxygen atoms (red) and their centers (green) highlighted. (**c**) Comparison of the oxygen centers between models with *J*_ind._ = 0.997 (green) and 0.975 (white).

For the models with the largest *J*_ind._ (i.e., better agreement between SAXS experiment, Fig. 6a), we evaluated the centers (green) of the four oxygen atoms (red) of each PACaL3 molecule and constructed a polyhedron by connecting thus obtained six centers as shown in Fig. 6b. Interestingly, the obtained six polyhedra are quite similar to the regular octahedron. On the other hand, for the models with the smallest *J*_ind._, the positions of the oxygen centers differ significantly from the regular octahedron (Fig. 6c). Consequently, when the micelle conformation determined by MD is in better agreement with the data, each PACaL3 molecule aligns in the same direction from the center to a vertex of the regular octahedron. This implies that the interface of PACaL3 takes the same spatial arrangement with that predicted by the Tammes problem.

## Summary

The present results indicate that a suitable combination of surfactant tail length, head volume, and sufficiently small *N*_*agg*_, as well as some rigidity of the building block is necessary to produce the “Platonic micelles”, which are monodisperse with a defined *N*_*agg*_ whose values are chosen from 6, 8, 12, and 32. The specific *N*_*agg*_ values is related to the mathematical Tammes problem, and because of the generality of this mathematical model, we presume that “the Platonic nature” may hold for any spherical micelles when *N*_*agg*_ is sufficiently small.

## Additional Information

**How to cite this article:** Fujii, S. *et al*. Platonic Micelles: Monodisperse Micelles with Discrete Aggregation Numbers Corresponding to Regular Polyhedra. *Sci. Rep.* **7**, 44494; doi: 10.1038/srep44494 (2017).

**Publisher's note:** Springer Nature remains neutral with regard to jurisdictional claims in published maps and institutional affiliations.

## Supplementary Material

Supplementary Information
