# Supplementary material for: Platonic Micelles: Monodisperse Micelles with Discrete Aggregation Numbers Corresponding to Regular Polyhedra
Source: Sci Rep. 2017 Mar 14;7:44494. doi: 10.1038/srep44494 (PMC5349551; doi:10.1038/srep44494)

Supplementary information

Platonic Micelles: Monodisperse Micelles with Discrete Aggregation Numbers Corresponding to Regular Polyhedra

Shota Fujii, Shimpei Yamada, Sakiko Matsumoto, Genki Kubo, Kenta Yoshida, Eri Tabata, Rika Miyake, Yusuke Sanada, Isamu Akiba, Tadashi Okobira, Naoto Yagi, Efstratios Mylonas, Noboru Ohta, and Hiroshi Sekiguchi and Kazuo Sakurai *

*Corresponding author. Email: sakurai@kitakyu-u.ac.jp

Table of Contents

Table S1. List of the calixarene-based amphiphilic molecules used in this study 2
Table S2. Symbols of physical parameters and their definitions 4
(S1) Hartley's micelle model 5
(S2) Synthesis procedures of calixarene-based lipids used in this study 6
(S3) Characterization of the micelles of QACaL*n* 11
(S4) A brief explanation of aFFF 14
(S5) The effects of pH and salt concentration on the aggregation behavior of the micelles composed of ECaL3 and QACaL5, respectively 15
(S6) Ishii's micelles: An example of continuous change in Nagg 19
(S7) How to calculate the weight fraction of the octamer to the unimer ($w.f.$) 21
(S8) FFF-MALS fractgrams for PEG*n*CaL5 micelles 22
(S9) Surfactine_CD spectra and SAXS data 23
(S10) Optimal configuration of N points on a sphere 24
(S11) Thermodynamics of spherical micelles 26
(S12) Computer chemistry 29
(S13) The Jaccard similarity coefficient 31
(S14) ^{1}H NMR spectra of calix[4]arene-based lipids 32

Table S1. List of the calixarene-based amphiphilic molecules used in this study

Chemical structures	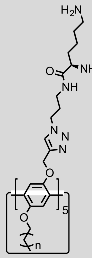	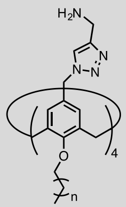			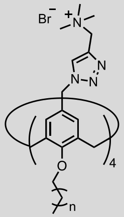							
Sample codes	KPiL4	PACaL*n*			QACaL*n*							
Reference	Published(1)	Published(2)			new							
Alkyl chain length	4	3	6	9	3	4	5				6	7
Solvent conditions	pH = 3.0 C_{NaCl} = 50mM	pH = 3.0 C_{NaCl} = 50mM			C_{NaCl} = 50 mM			C_{NaCl} = 100 mM	C_{NaCl} = 200 – 300 mM	C_{NaCl} > 400 mM	C_{NaCl} = 50 mM	C_{NaCl} = 50 mM
CMC / μM	100	110	4.0	2.9	—							
Morphology*	S	S	S	C	S	S	S	S	S	M	S	S
N_{agg} (SAXS)	2.0	6.0	12	—	8.0	8.0	12	12	20	—	12	20
N_{agg} (MALS)	2.0	6.0	—	—	8.0	8.0	12	—	—	—	12	20
N_{agg} (AUC)	—	6.0	12	—	8.0	8.0	12	—	—	—	12	20
M_W/M_N	1.00**	1.01	—	—	1.03	1.00	1.00	—	—	—	1.00	1.01
M_Z/M_W	—	1.07	1.50	—	1.07	1.02	1.02	—	—	—	1.01	1.03

Chemical structures	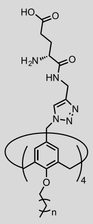			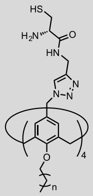	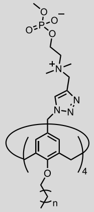		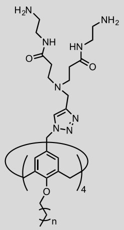	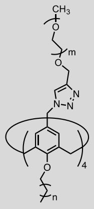	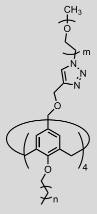	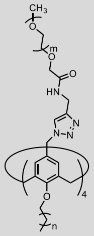	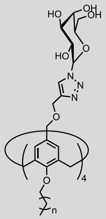	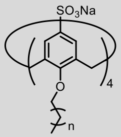	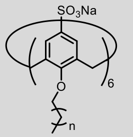
Sample codes	ECaL3			CCaL3	CPCaL*n*		G1CaL3	PEG*n*CaL5			GalCaL3	SC4AH	SC6AH
Reference	new			Published(3)	Published(4)		new	new			new	new	Published(5)
Alkyl chain length	3			3	3	6	3	5			3	5	5
Solvent conditions	pH = 3.2	pH = 7.5	pH = 10	pH = 3.0	C_{NaCl} = 150 mM		C_{NaCl} = 50 mM	C_{NaCl} = 50 mM			C_{NaCl} = 50 mM	C_{NaCl} = 100 mM	C_{NaCl} = 0 mM
CMC / μM	4.4	1.0	1.8	—	1.8	0.4	—	—			—	—	500
Morphology*	S	C	S	S	S		S	S			V	S	S
N_{agg} (SAXS)	6.0	—	12	12	—	—	8	20	12	—	—	—	—
N_{agg} (MALS)	6.0	—	12	12	8.0	32	8	20	12	3.6	—	12	6.0
N_{agg} (AUC)	—	—	—	—	—	—	—	—	—	—	—	12	—
M_W/M_N	1.04	—	1.00	1.00	1.04	1.00	1.03	1.00	1.01	1.13	—	1.00	—
M_Z/M_W	—	—	—	—	—	—	—	—	—	—	—	1.04	—

(1) Nishimura et al. A bimolecular micelle constructed from amphiphilic pillar[5]arene molecules. *Chem. Commun*., 2013, 49, pp 3025-3054
(2) Fujii et al. A Stimulus-Responsive Shape-Persistent Micelle Bearing a Calix[4]arene Building Block: Reversible pH-Dependent Transition between Spherical and Cylindrical Forms. *Langmuir*, 2012, 28, pp 3092-3101
(3) Fujii et al. Synthesis and Characterization of a Calix[4]arene Amphiphilie Bearing Cysteine and Uniform Au Nanoparticle Formation Templated by its Four Cysteine Moieties. *Langmuir*, 2013, 29, pp 13666-13675
(4) Fujii et al. Micelles consisting of choline phosphate-bearing Calix[4]arene lipids. *Soft Matter*, 2014, 10, pp 8216-8223
(5) Shinkai et al. Hexasulfonated calix[6]arene derivatives: a new class of catalysts, surfactants, and host molecules. *J. Am. Chem. Soc.*, 1986, 108, pp 2409-2416

*: S, C, V, and M are sphere, cylinder, vesicle, and mixture of various micellar shapes, respectively.

**: Due to the effect of the cross flow in FFF system, the micellar fractogram shows the presence of unimer and trimer as well as dimer. The micellar monodispersity is determined from the SAXS profile demonstrating the first sharp minimum, which indicates monodisperse micelle.

Table S2. Symbols of physical parameters and their definitions

N_{agg}	Aggregation number
$D(N)$	Coverage ratio
a_e (packing parameter principle)	Equilibrium area for molecule at the aggregate interface
V (packing parameter principle)	Volume of surfactant tails
h (packing parameter principle)	Length of surfactant tails
n_c	The number of carbons in an alkyl chain
M_n	Number averaged molecular weight (gmol^{-1})
M_w	Weight averaged molecular weight (gmol^{-1})
r (analytical ultracentrifugation)	Distance from rotating center
$C(r)$ (analytical ultracentrifugation)	The concentration at r after reached equilibrium
$I(q)$ (small angle X-ray scattering)	Scattering intensity
q (small angle X-ray scattering)	Magnitude of scattering vector (nm^{-1})
C_{NaCl}	NaCl concentration (mM)
φ	Water volume percent
w.f.	Weight fraction ratio of the octamer to the monomer
σ	The contact free energy per unit area

(S1) Hartley's micelle model

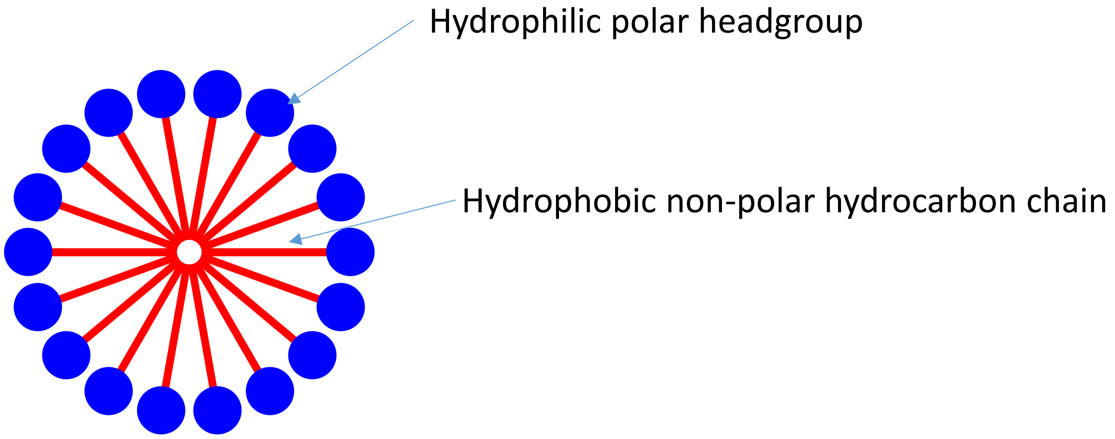

Hartley was first to assume that a spherical, core–shell shape was the most likely explanation for the concentration dependence of surfactant solution conductivity with the hydrophobic hydrocarbon chains occupying the core and the hydrophilic polar head groups forming the shell.

(S2) Synthesis procedures of calixarene-based lipids used in this study

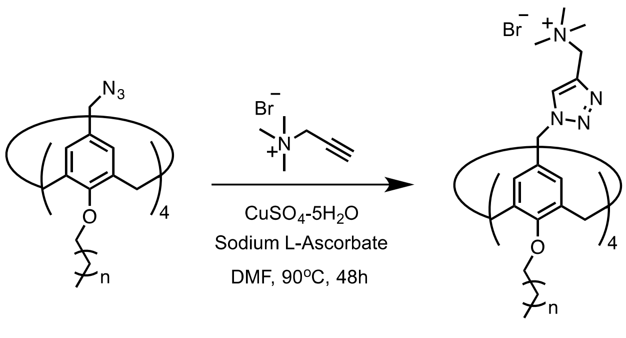

Synthesis of quaternary ammonium bromides bearing calix[4]arene lipid with propyl tails ***(QACaL3).*** N,N,N-trimethyl-2-propyn-1-ammmonium bromide (0.280 g, 1.57 mmol), copper(II) sulfate pentahydrate (4.10 mg, 16.4 μmol), and sodium ascorbate (32.1 mg, 0.162 mmol) were dissolved in dry DMF (10 mL), and then a solution of azide bearing calix[4]arene derivative (0.127 g, 0.156 mmol) in dry DMF (5 mL) was added to the mixture. The reaction mixture was stirred for 48 h at 90 °C. The mixture was cooled to room temperature, and then concentrated at reduced pressure. The crude product was purified by reversed phase silica gel column chromatography (water), which afforded a brown solid (0.1863 g, 0.121 mol, 76%). ^{1}H NMR (500 MHz, $CDCl_3$): δ(ppm) = 8.46 (s, 4H), 6.71 (s, 8H), 5.40 (s, 8H), 4.75 (s, 8H), 4.46 (d, J = 10.0 Hz, 4H), 3.83 (t, J = 7.50 Hz, 8H), 3.21 (d, 4H), 3.17 (s, 9H), 1.90 (m, 8H), 1.00 (t, J = 7.5 Hz 12H). ESI–MS ($M^{2+}/2$): calcd for $C_{68}H_{100}Br_2N_{16}O_4$ 682.32, found 682.34.

Synthesis of quaternary ammonium bromides bearing calix[4]arene lipid with butyl tails ***(QACaL4).*** QACaL4 was synthesized using a similar procedure described for QACaL3. Yield: 0.168 g, 0.107 mol, 20%. ^{1}H NMR (500 MHz, $CDCl_3$): δ(ppm) = 8.41 (s, 4H), 6.69 (s, 8H), 5.37 (s, 8H), 4.68 (s, 8H), 4.38 (d, J = 10.0 Hz, 4H), 3.62 (t, J = 7.50 Hz, 8H), 3.17 (d, 4H), 3.09 (s, 9H), 1.83 (m, 8H), 1.42 (m, 8H), 0.94 (t, J = 7.5 Hz 12H). ESI–MS ($M^{2+}/2$): calcd for $C_{72}H_{108}Br_2N_{16}O_4$ 710.35, found 710.37.

Synthesis of quaternary ammonium bromides bearing calix[4]arene lipid with pentyl tails ***(QACaL5).*** QACaL5 was synthesized using a similar procedure described for QACaL3. Yield: 0.631 g, 0.386 mol, 93%. ^{1}H NMR (500 MHz, $CDCl_3$): δ(ppm) = 8.38 (s, 4H), 6.65 (s, 8H), 5.34 (s, 8H), 4.68 (s, 8H), 4.37 (d, J = 10.0 Hz, 4H), 3.61 (t, J = 7.50 Hz, 8H), 3.16 (d, 4H), 3.09 (s, 9H), 1.84 (m, 8H), 1.35 (m, 8H), 0.89 (t, J = 7.5 Hz 12H). ESI–MS ($M^{2+}/2$): calcd for $C_{76}H_{116}Br_2N_{16}O_4$ 738.39, found 738.41.

Synthesis of quaternary ammonium bromides bearing calix[4]arene lipid with hexyl tails ***(QACaL6).*** QACaL6 was synthesized using a similar procedure described for QACaL3. Yield: 0.241 g, 0.142 mol, 80%. ^{1}H NMR (500 MHz, $CDCl_3$): δ(ppm) = 8.47 (s, 4H), 6.73 (s, 8H), 5.41

(s, 8H), 4.75 (s, 8H), 4.43 (d, J = 10.0 Hz, 4H), 3.87 (t, J = 7.50 Hz, 8H), 3.17 (d, 4H), 3.16 (s, 9H), 1.91 (m, 8H), 1.38 (m, 8H), 0.94 (t, J = 7.5 Hz 12H). ESI–MS ($M^{2+}/2$): calcd for $C_{80}H_{124}Br_2N_{16}O_4$ 766.42, found 766.44.

Synthesis of quaternary ammonium bromides bearing calix[4]arene lipid with heptyl tails ***(QACaL7).*** QACaL7 was synthesized using a similar procedure described for QACaL3. Yield: 0.123 g, 0.0705 mol, 86%. ^{1}H NMR (500 MHz, $CDCl_3$): δ(ppm) = 8.48 (s, 4H), 6.71 (s, 8H), 5.40 (s, 8H), 4.78 (s, 8H), 4.43 (d, J = 15.0 Hz, 4H), 3.87 (t, J = 7.50 Hz, 8H), 3.24 (d, 4H), 3.19 (s, 9H), 1.91 (m, 8H), 1.35 (m, 8H), 0.91 (t, J = 7.5 Hz 12H). ESI–MS ($M^{2+}/2$): calcd for $C_{84}H_{132}Br_2N_{16}O_4$ 794.45, found 794.47.

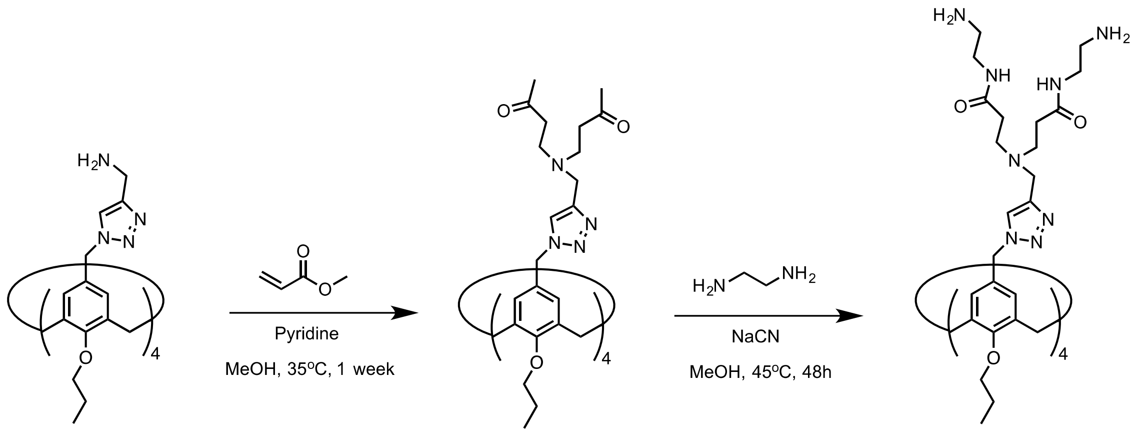

G1CaL3

Synthesis of polyamidoamines (G1 PAMAM) bearing calix[4]arene lipid with propyl tails ***(G1CaL3).*** Primary amine bearing calix[4]arene lipid with propyl tails (PACaL3) (0.542 g, 0.525 mmol), pyridine (0.373 g, 5.25 mmol), and methyl acrylate (18.08 g, 0.21 mol) were dissolved in dry methanol (10 mL) at room temperature. The reaction mixture was allowed to warm to 35 °C and stirred for 1 week. The mixture was cooled to room temperature, and then concentrated at reduced pressure. The crude product was mixed with sodium cyanide (2.20 mg, 45.2 μmol) in dry methanol (10 mL). Ethylene diamine (9.51 g, 0.158 mol) was added into the reaction mixture, and then allowed to warm to 45 °C and stirred for 48 h. The mixture was cooled to room temperature, and then the product was purified with Spectra/Por Float-A-Lyzer (cellulose membrane; cut off 1 kDa) for 5 days (0.435 g, 0.224 mmol, 43%). ^{1}H NMR (500 MHz, methanol-d4): δ(ppm) = 7.63 (s, 4H), 6.63 (s, 8H), 5.30 (s, 8H), 4.44 (d, J = 15.0 Hz, 4H), 3.84 (t, 7.50 Hz, 8H), 3.79 (s, 8H), 3.22 (t, J = 7.50 Hz, 16H), 2.76–2.69 (m, 32H), 2.41 (t, J = 7.50 Hz, 16H), 1.92 (m, 8H), 1.00 (t, J = 7.50 Hz, 12H). ESI–MS ($M^{4+}/4$ + 4H): calcd for $C_{97}H_{158}N_{32}O_{12}$ 491.07, found 491.57.

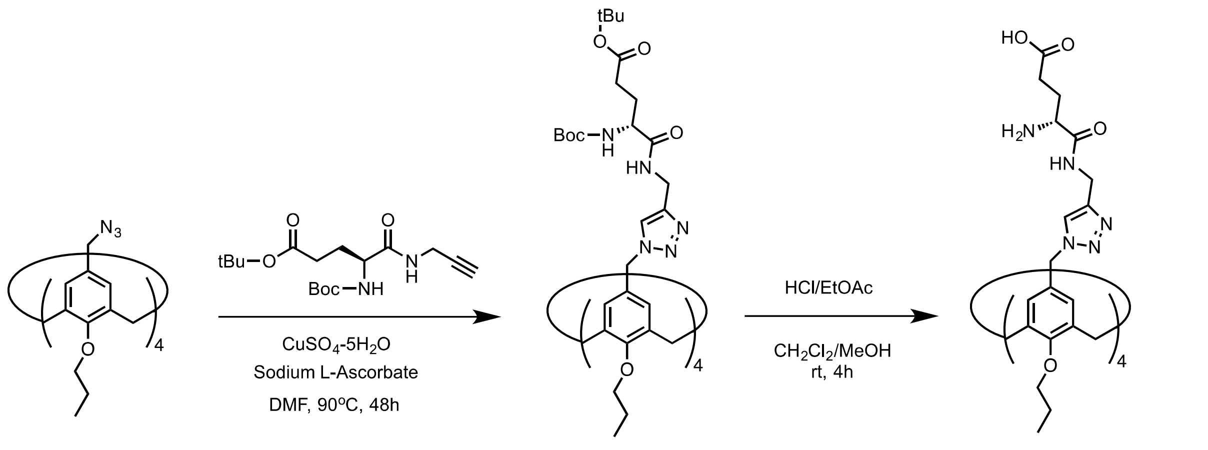

ECaL3

Synthesis of protected glutamic acids bearing calix[4]arene lipid with propyl tails. N-(2-propynyl)-Boc-L-Glu(OtBu) (0.810 g, 2.38 mmol), copper (II) sulfate pentahydrate (11.9 mg, 47.6 μmol), and sodium ascorbate (28.3 mg, 0.143 mmol) were dissolved in dry DMF (10 mL), and then a solution of azide bearing calix[4]arene derivative (0.391 g, 0.476 mmol) in dry DMF (5 mL) was added to the mixture. The mixture was stirred at 80 °C for 24 h, and then cooled to room temperature. Water was then added, and the reactant was extracted with EtOAc. The organic layer was washed three times with saturated NaCl solution and dried over $MgSO_4$. The solution was evaporated to dryness, and the residue was purified by flash chromatography (10:1 EtOAc/Methanol), which afforded a brown solid (0.798 g, 0.367 mol, 77%). ^1H NMR (400 MHz, $CDCl_3$): δ (ppm) = 7.68 (br, 4H), 7.54 (s, 4H), 6.45 (s, 8H), 5.62 (br, 4H), 5.16 (s, 8H), 4.59–4.44 (m, 8H), 4.36 (d, J = 13.2 Hz, 4H), 4.18 (br, 4H), 3.78 (t, J = 7.44 Hz 8H), 3.06 (d, J = 13.4 Hz, 4H), 2.28 (br, 8H), 2.08–1.84 (m, 8H), 1.87 (m, 8H), 1.41–1.38 (m, 18H), 0.96 (t, J = 7.36 Hz, 12H).

*Synthesis of glutamic acids bearing calix[4]arene lipid with propyl tails (**ECaL3**).* A solution of the protected glutamic acids bearing calix[4]arene derivative (0.362 g, 0.167 mol) was treated with 4N HCl/EtOAc for 1 h. The solvent was evaporated, and the residue was washed with CH_2Cl_2 and EtOAc. ECaL3 was obtained as a white solid (0.253 mg, 0.163 mmol, 98 %). ^1H NMR (400 MHz, methanol-d4): δ = 8.02 (s, 4H), 6.67 (s, 8H), 5.33 (m, 8H), 4.55 (m, 8H), 4.43 (d, J = 13.2 Hz, 4H), 3.99 (t, J = 6.32 Hz, 4H), 3.83 (t, J = 7.28 Hz, 8H), 3.15 (d, J = 13.5 Hz, 4H), 2.47 (m, 8H), 2.14 (m, 8H), 1.92 (m, 8H), 1.00 (t, J = 7.48 Hz, 12H). ^{13}C NMR (100 MHz, methanol-d_4): δ (ppm) = 172.7, 168.5, 156.9, 135.4, 128.3, 128.2, 124.2, 76.8, 54.3, 52.4, 51.1, 33.9, 30.3, 28.7, 26.1, 23.0, 9.33.

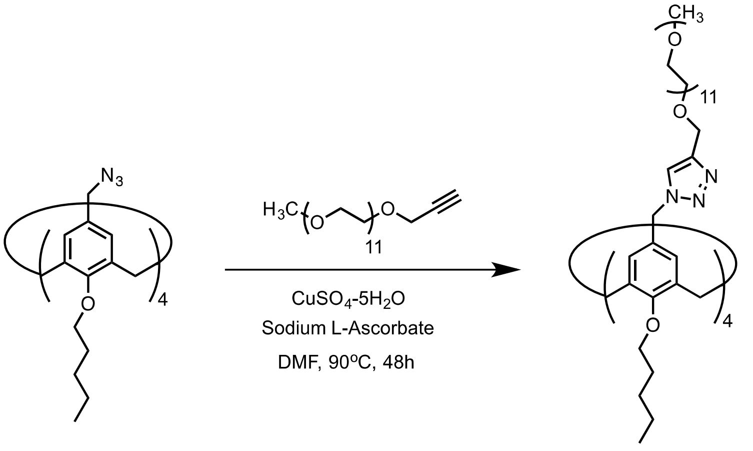

PEG550CaL5

*Synthesis of polyethylene glycol (PEG) (M_W = 550 g/mol) bearing calix[4]arene lipid with pentyl tails(**PEG550CaL5**).* A PEG derivative (0.163 g, 0.295 mmol), copper (II) sulfate pentahydrate (2.20 mg, 8.81 μmol), and sodium ascorbate (3.70 mg, 18.7 μmol) were dissolved in dry DMF (10 mL), and then a solution of azide bearing calix[4]arene derivative (54.6 mg, 0.0590 mmol) in dry DMF (5 mL) was added to the mixture. The mixture was stirred at 90 °C for 48 h, and then cooled to room temperature. The crude product was purified with Spectra/Por Float-A-Lyzer (cellulose membrane; cut off 1 kDa) for 5 days (13.7 mg, 0.0437 mmol, 74%). ^{1}H NMR (500 MHz, $CDCl_3$): δ (ppm) = 7.46 (s, 4H), 6.55 (s, 8H), 5.22 (s, 8H), 4.65 (s, 8H), 4.38 (d, J = 10.0 Hz, 4H), 3.84 (t, J = 7.50 Hz, 8H), 3.70–3.51 (m, 4H), 3.38 (s, 12H), 3.08 (d, J = 15.0 Hz 4H), 1.88–1.85 (m, 8H), 1.38–1.36 (m, 16H), 0.93 (t, J = 7.50 Hz, 12H).

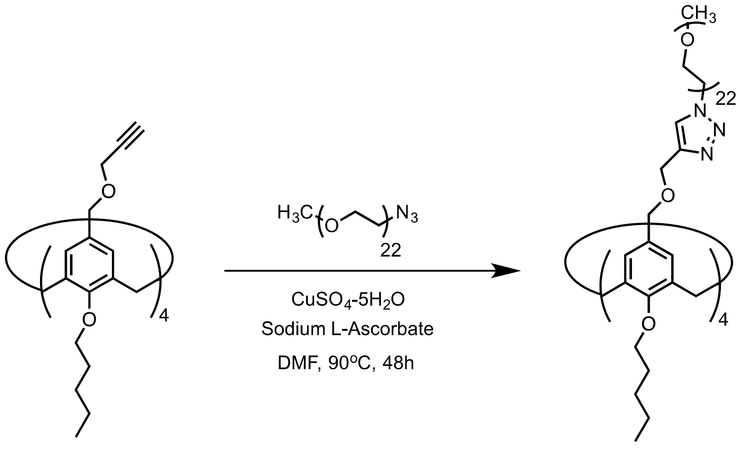

PEG1kCaL5

*Synthesis of polyethylene glycol (PEG) (M_W = 1000 g/mol) bearing calix[4]arene lipid with pentyl tails (**PEG1kCaL5**).* A PEG derivative (0.606 g, 0.590 mmol), copper (II) sulfate pentahydrate (2.00 mg, 8.01 μmol), and sodium ascorbate (6.10 mg, 30.8 μmol) were dissolved

in dry DMF (10 mL), and then a solution of propargyl calix[4]arene derivative (91.7 mg, 0.0939 mmol) in dry DMF (5 mL) was added to the mixture. The mixture was stirred at 90 °C for 48 h, and then cooled to room temperature. The crude product was purified with Spectra/Por Float-A-Lyzer (cellulose membrane; cut off 3.5 kDa) for 7 days (0.328 g, 0.0646 mmol, 69%). ^{1}H NMR (500 MHz, $CDCl_3$): δ (ppm) = 7.69 (s, 4H), 6.60 (s, 8H), 4.54–4.51 (m, 8H), 4.40 (d, J = 15.0 Hz, 4H), 4.22 (s, 8H), 3.86 (t, J = 7.50 Hz, 8H), 3.68–3.49 (m, #H), 3.39 (s, 12H), 3.11 (d, J = 10.0 Hz 4H), 1.89–1.86 (m, 8H), 1.37–1.36 (m, 16H), 0.93 (t, J = 7.50 Hz, 12H).

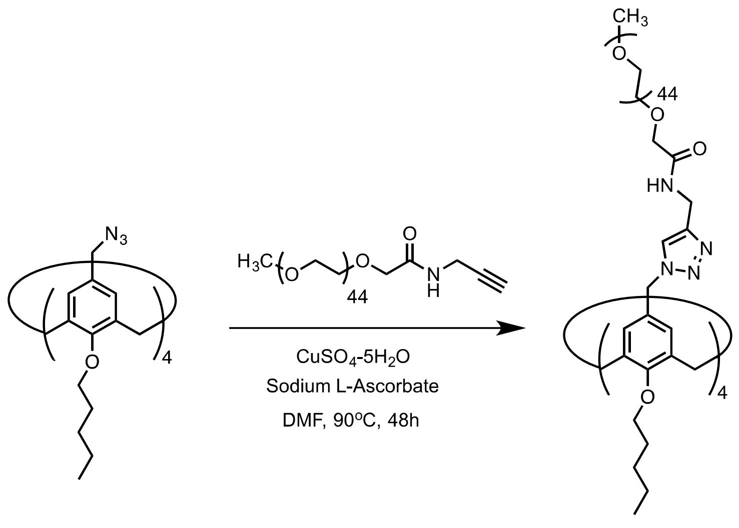

*Synthesis of polyethylene glycol (PEG) (M_W = 2000 g/mol) bearing calix[4]arene lipid with pentyl tails (**PEG$_{2k}$CaL5**).* A PEG derivative (0.303 g, 0.153 mmol), copper (II) sulfate pentahydrate (2.5 mg, 10.0 μmol), and sodium ascorbate (9.20 mg, 46.4 μmol) were dissolved in dry DMF (10 mL), and then a solution of azide bearing calix[4]arene derivative (30.5 mg, 0.0330 mmol) in dry DMF (5 mL) was added to the mixture. The mixture was stirred at 90 °C for 48 h, and then cooled to room temperature. The crude product was purified with Spectra/Por Float-A-Lyzer (cellulose membrane; cut off 3.5 kDa) for 7 days (0.259 g, 0.0293 mmol, 88%). ^{1}H NMR (500 MHz, $CDCl_3$): δ (ppm) = 7.57 (t, J = 5.00 Hz, 4H), 7.44 (s, 4H), 6.56 (s, 8H), 5.20 (s, 8H), 4.53 (d, J = 5.00 Hz, 8H), 4.40 (d, J = 15.0 Hz, 4H), 3.98 (s, 8H), 3.86 (m, 8H), 3.79–3.49 (m, #H), 3.38 (s, 12H), 3.10 (d, J = 10.0 Hz, 4H), 1.87 (m, 8H), 1.37 (m, 16H), 0.93 (t, J = 7.50 Hz, 12H).

(S3) Characterization of the micelles of QACaL*n*

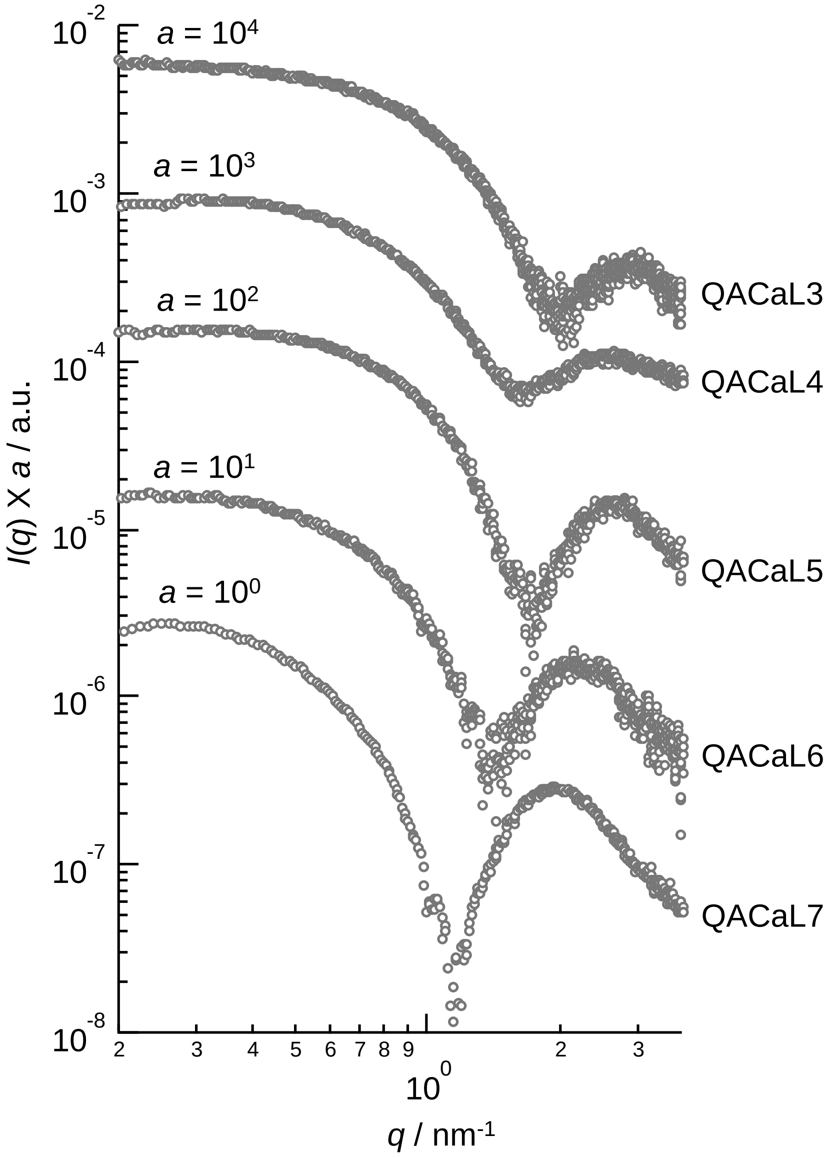

Figure S3-1. SAXS profiles of QACaL*n* micelles in 50 mM NaCl aqueous solution.

Molecular weight deteminations from SAXS and aFFF/MALS.

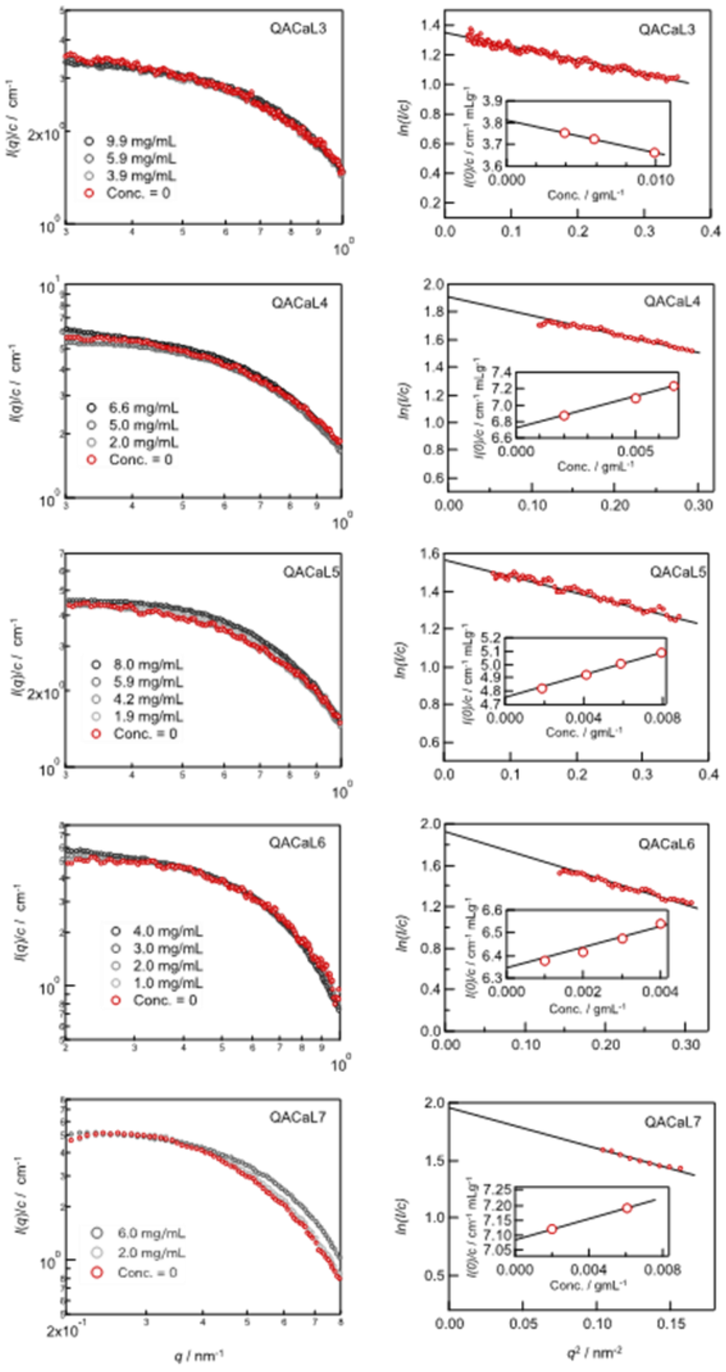

Figure S3-2. Left side: $I(q)/c$ as a function of q for different QACaLn concentrations in 50 mM NaCl solution. The extrapolated values at infinite dilution for each q are shown by the red markers. Right side: The Guinier plots (i.e., ln $I(q)/c$ versus q^2) constructed from the extrapolated intensities. The inserts show the concentration dependence of the $I(0)/c$ values. The micellar molar mass determined from the intercept values at $q = 0$ and concentration = 0 agree with each other

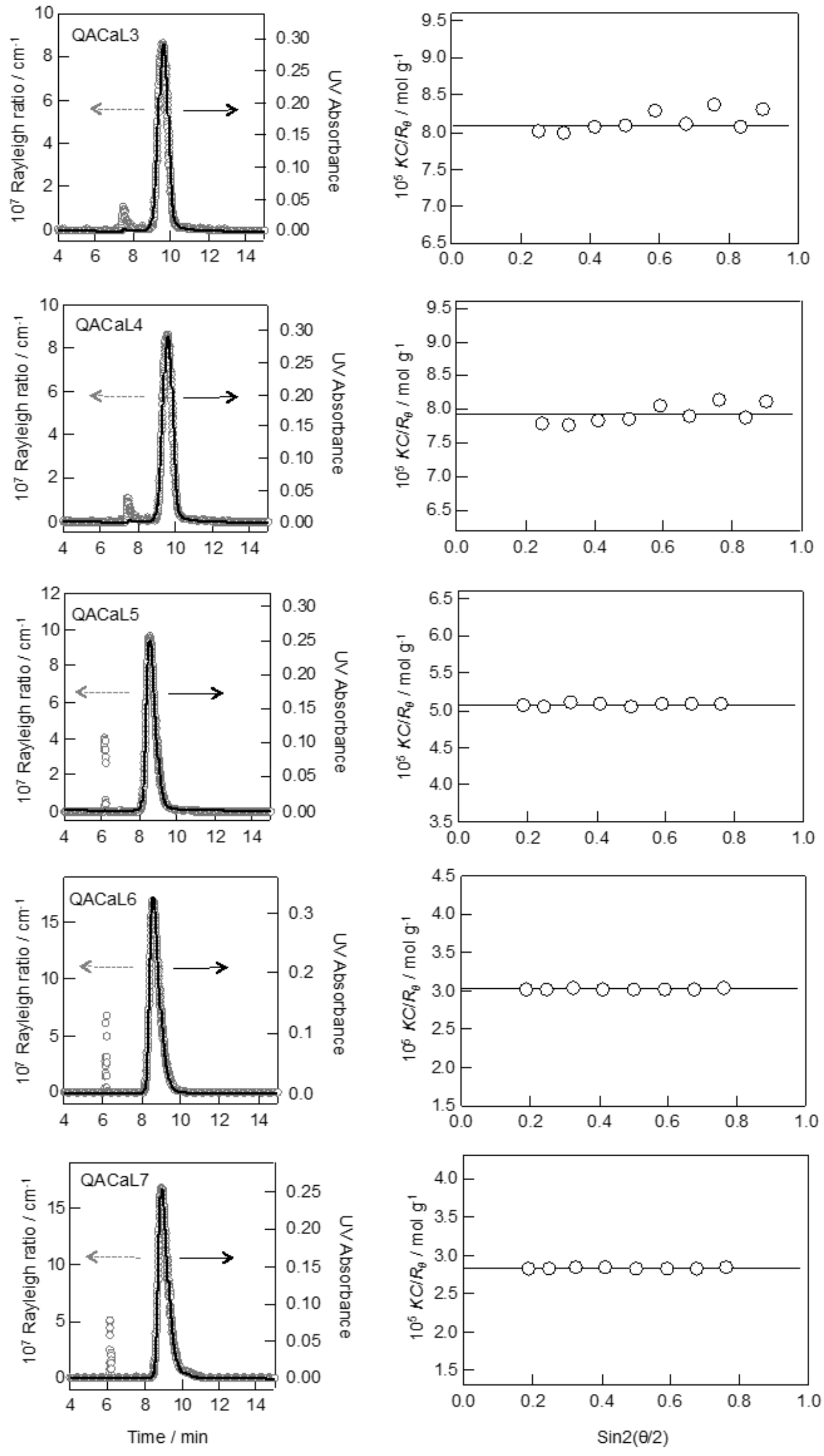

Figure S3-3. S3-3. Left side: FFF-MALS fractograms of QACaL*n* micelles measured by LS at 90° (circle) and UV spectrophotometry at 270 nm (line) in aqueous 150 mM NaCl. Right side: The Zimm plots for QACaL*n* micelles at the top of the UV peaks.

(S4) A brief explanation of aFFF

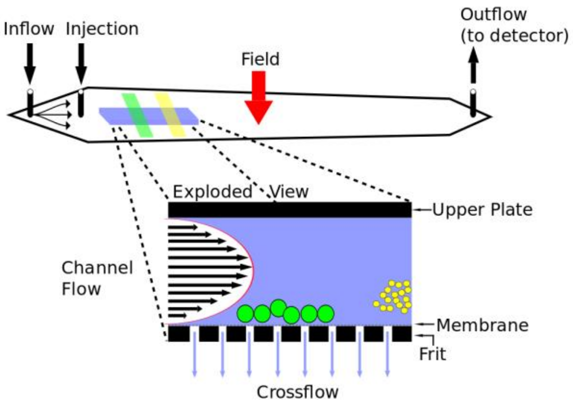

From Wikipedia

Asymmetrical field flow fractionation (aFFF) is a method for separating nanoparticles, polymers, and proteins with combined channel and cross flows. The cross flow is induced by the constant exiting of the carrier liquid by way of a semipermeable wall on the bottom of the channel. We used an Eclipse 3+ separation system (Wyatt Technology Europe, Dernbach, Germany), which was sequentially connected to a Dawn Heleos II multi-angle static light scattering (SLS) detector (Wyatt Technology) and an Optilab rEX DSP differential refractive index (RI) detector (Wyatt Technology) operating at a wavelength of 658 nm. A Wyatt channel (Eclipse 3 channel LC) with a tip-to-tip length of 17.4 cm and a nominal thickness of 250 μm was used. A Nadir cellulose membrane (10 kDa LC) was attached on the bottom of the channel. Samples were injected into the system with the injected solution positioned at a specified area on the membrane before fractionation. This process is called “sample focusing”. After focusing, two flows were applied: the laminar (or channel) flow that carries the sample through the separation chamber with the separation field applied perpendicular to the channel creating the cross flow. The cross flow rate can be held constant or systematically changed; normally it is gradually decreased. The cross flow pushes the particles toward the bottom of the channel where a membrane filter is attached. The extent to which particles can diffuse back into the channel is determined by the diffusion coefficient of the particles and the rate of the cross flow.

Specific refractive index increments were determined for each sample with a DRM-1021 differential refractometer (Otuska Electronics). The values obtained were used to determine the weight-averaged molar masses (Mw) and radii of gyration (R_g). The hydrodynamic radii (R_H) were determined by DLS (Malvern Instruments, Malvern, UK).

(S5) The effects of pH and salt concentration on the aggregation behavior of the micelles composed of ECaL3 and QACaL5, respectively.

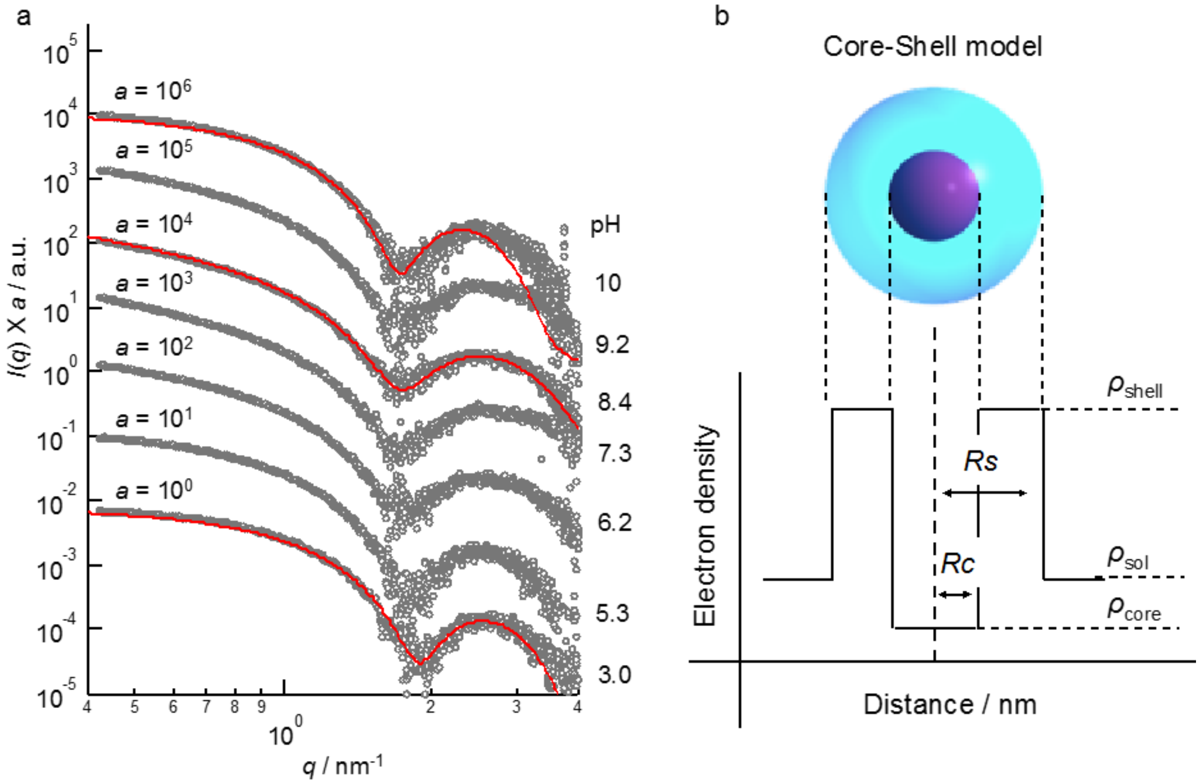

Figure S5-1. (a) SAXS profiles for ECaL3 micelles in 50 mM NaCl at different pH values. The SAXS profiles were fitted to a core–shell spherical model (b) as follows:

$$I(q) \sim \left(\frac{V_c(\rho_c - \rho_s)(\sin(qR_c) - qR_c cos(qR_c))}{(qR_c)^3} + \frac{V_s(\rho_s - \rho_{sol})(\sin(qR_s) - qR_s cos(qR_s))}{(qR_s)^3} \right)^2$$

where R_c and R_s are the thicknesses of the core and shell, respectively, andρ_c,ρ_s, and ρ_{sol} are the electron densities of the core, shell, and solvent, respectively. According to our previous paper, ρ_{sol} and ρ_c are 334 and 270 e nm^{-3}, respectively. The solid red lines in Figure S5-1 represent the calculated values, which agree with the data.

Table S3. SAXS Fitting Parameters for ECaL3 Micelles at Different pHs

pH	R_g / nm	Model	R_C /nm	R_S /nm	R_S - R_C /nm	σ / R_S	ρ_C / e nm^{-1}	ρ_S / e nm^{-1}
3.0	1.64	sphere	0.700	2.15	1.45	0.06	270	400
8.3	—	infinite cylinder	0.420	1.80	1.38	0.05	270	365
10	1.82	sphere	0.700	2.38	1.68	0.05	270	400

R_C: core size, R_S: shell size, ρ_C: electron density of the core, ρ_S: electron density of the shell, σ: standard deviation

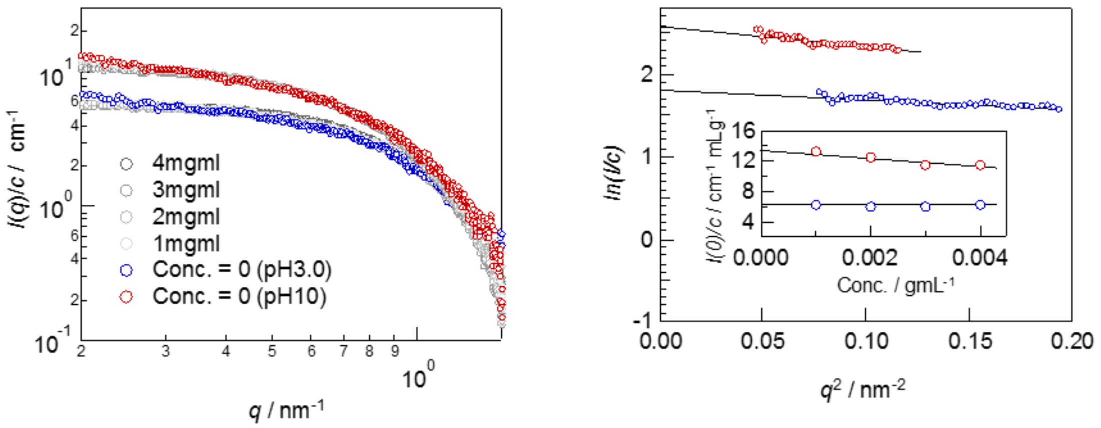

Figure S5-2. Left side: $I(q)/c$ as a function of q for different ECaL3 concentrations in 50 mM NaCl solution at pH = 3.0 and 10. The extrapolated values at infinite dilution for each q are shown by the red markers. Right side: The Guinier plot (i.e., ln $I(q)/c$ versus q^2) constructed from the extrapolated intensities. The insert show concentration dependence of the $I(0)/c$ values. The micellar molar mass determined from the intercept values at q = 0 and concentration = 0 agree with one another.

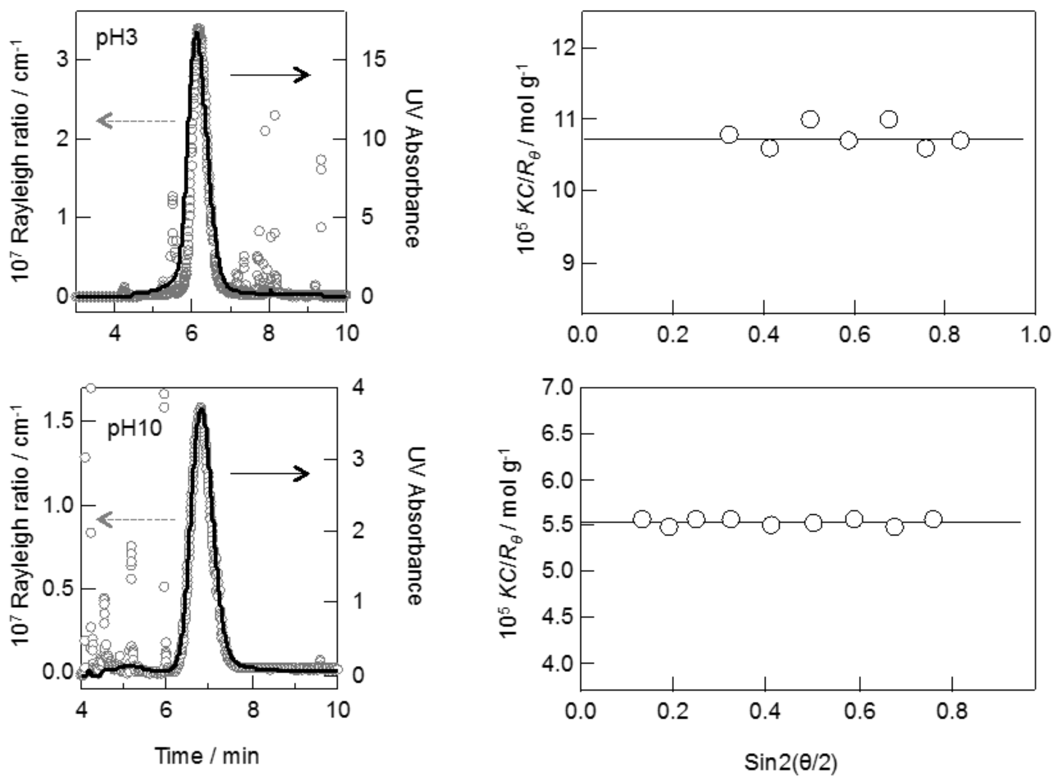

Figure S5-3. Left side: FFF-MALS fractogram of ECaL3 micelles at pH = 3.0 and pH = 10 measured by LS at 90° and UV at 270 nm. Right side: The Zimm plots for ECaL3 micelles at the top of the UV peaks.

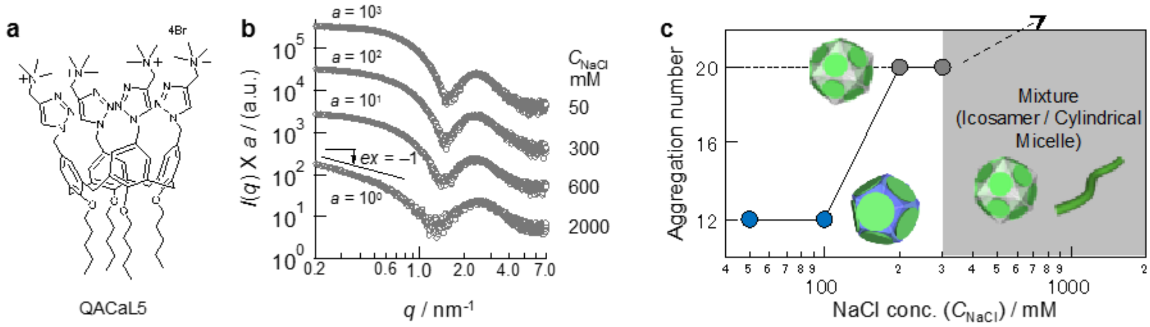

Figure S5-4. Chemical structure of QACaL5 (a). The NaCl concentration (C_{NaCl}) dependence of SAXS for QACaL5 (b) and N_{agg} is plotted against C_{NaCl} (c). With an increase of C_{NaCl}, N_{agg} increases from 12 to 20, both showing monodispersity. At $C_{\mathrm{NaCl}} > 300\,\mathrm{mM}$, the solution contains a mixture of icosamers and cylinders and N_{agg} approaches 40 at C_{NaCl} = 2000 mM and continuously increases with the increase of C_{NaCl}. The paper about the effects of salt concentration on the aggregation behavior of QACaL5 micelles is submitted to Chemistry Letters.

(S6) Ishii's micelles: An example of continuous change in Nagg

Accepted by *Polymer Journal* in 2106 May

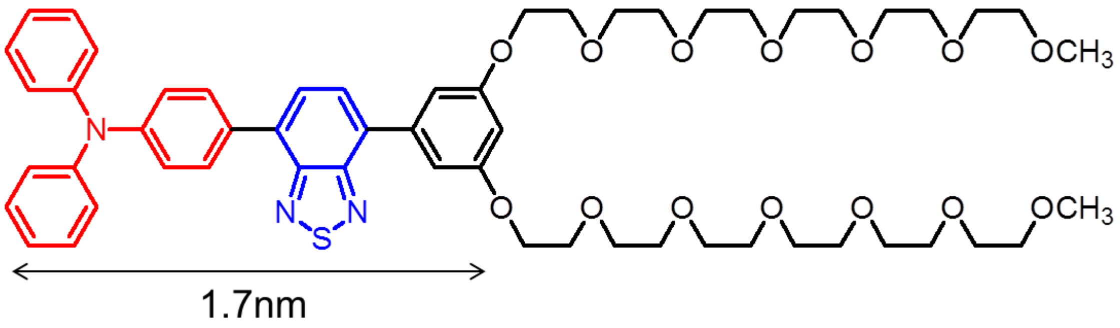

Figure S6-1. The chemical structure of the amphiphilic benzothiadiazole-triphenylamine molecule (BT-lipid)

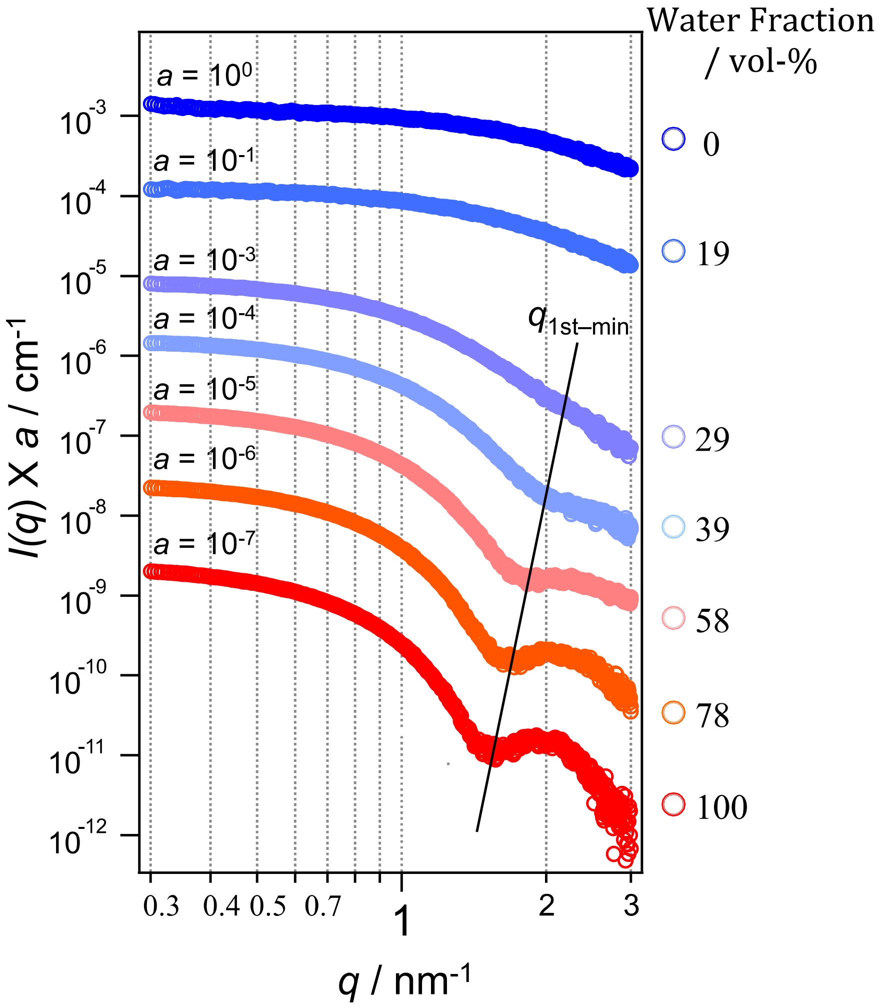

Figure S6-2. The water composition dependence of SAXS profiles for BT-lipid. SAXS measurements were performed at BL-40B2 of SPring-8 and the absolute X-ray scattering intensity was determined using the absolute scattering intensity of water of 1.632 × 10^{-2} cm^{-1}.

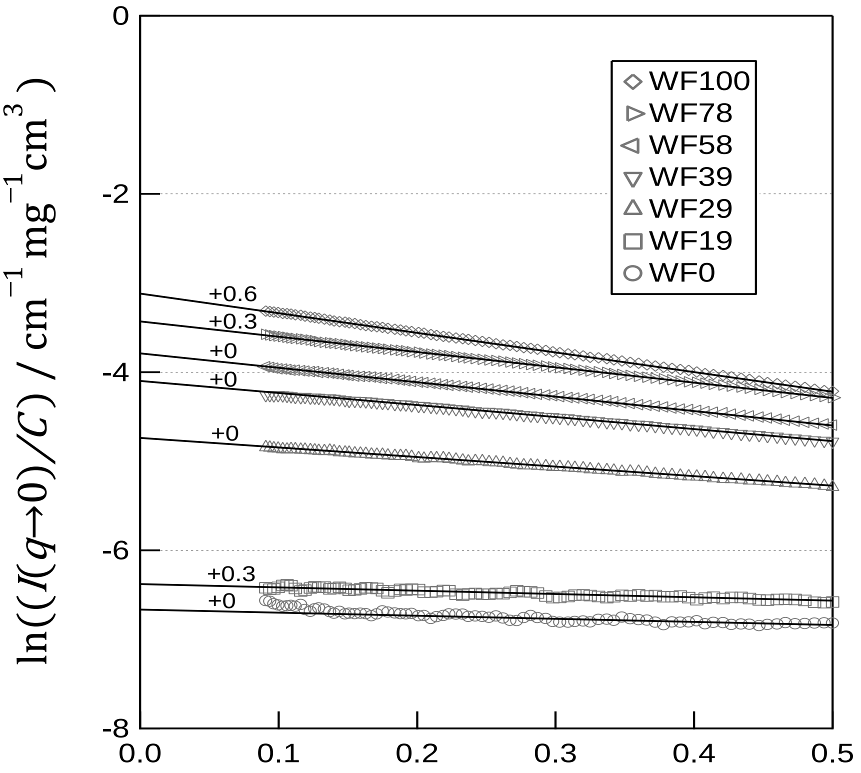

Figure S6-3. Guinier plot of BT-lipid with various water/methanol compositions.

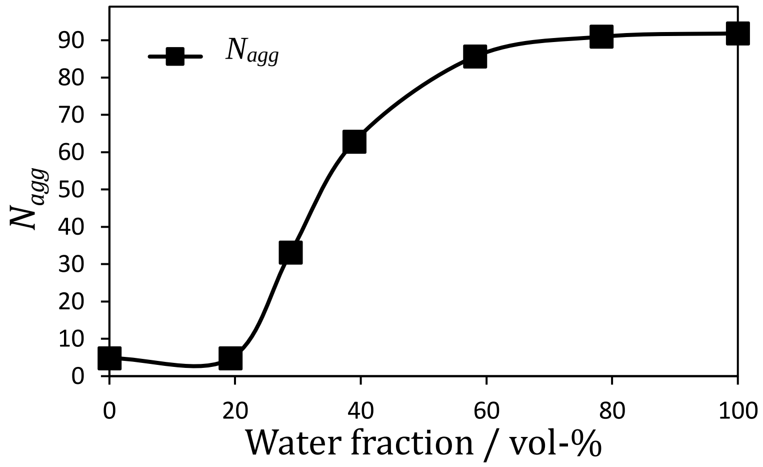

Figure S6-4. Water fraction dependence of N_{agg} of BT-lipid.

Ref. Polymer Journal accepted.

(S7) How to calculate the weight fraction of the octamer to the unimer ($w.f.$)

$$I = K\ V\ C$$

I :scattering intensity

K: instrument constant

V: scattering volume

C: weight concentration

Assuming that there are only octamer and unimer existing in solution

$$I = K\ V_{uni}\ C_{uni} + K\ V_{oct} C_{oct}$$

$$i_{uni} = K\ V_{uni}\ , i_{oct} = K\ V_{oct}$$

$$I = i_{uni}\ C_{uni} + i_{oct}\ C_{oct}$$

$$C_{uni} + C_{oct} = C_0$$

This can be regarded as a weight fraction.

$$C_{uni} + C_{oct} = 1$$

$$C_{uni} = 1 - C_{oct}$$

then

$$I = i_{uni}\ (1 - C_{oct}) + i_{oct}\ C_{oct}$$

(S8) FFF-MALS fractgrams for PEG*n*CaL5 micelles

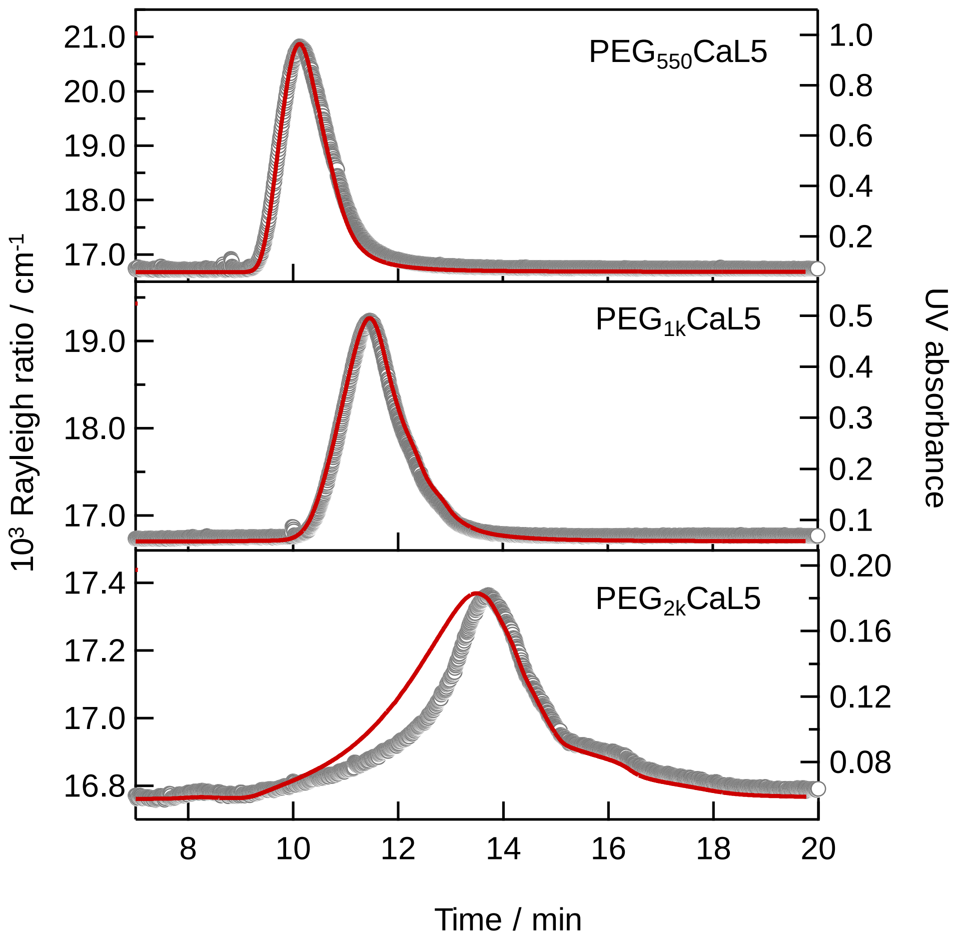

Figure S8. FFF-MALS fractogram of PEG*n*CaL5 micelles in 50 mM NaCl aqueous solution measured with LS at 90° and UV at 270 nm

(S9) Surfactine_CD spectra and SAXS data

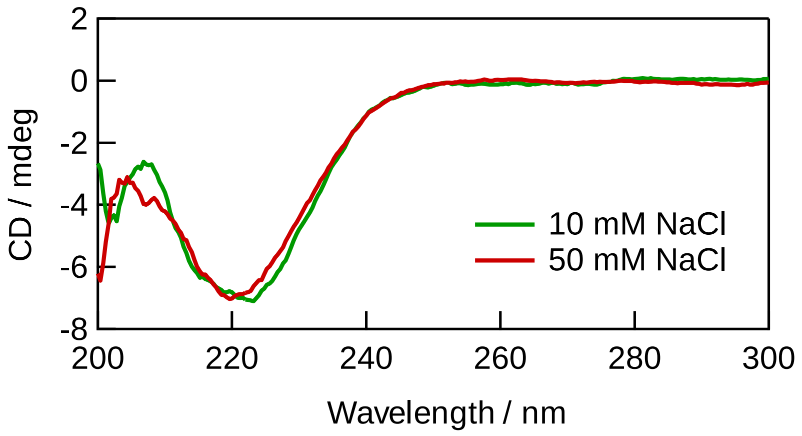

Figure S9-1. CD spectra of the micelles consisting of surfactine in 10 or 100 mM NaCl aqueous solution.

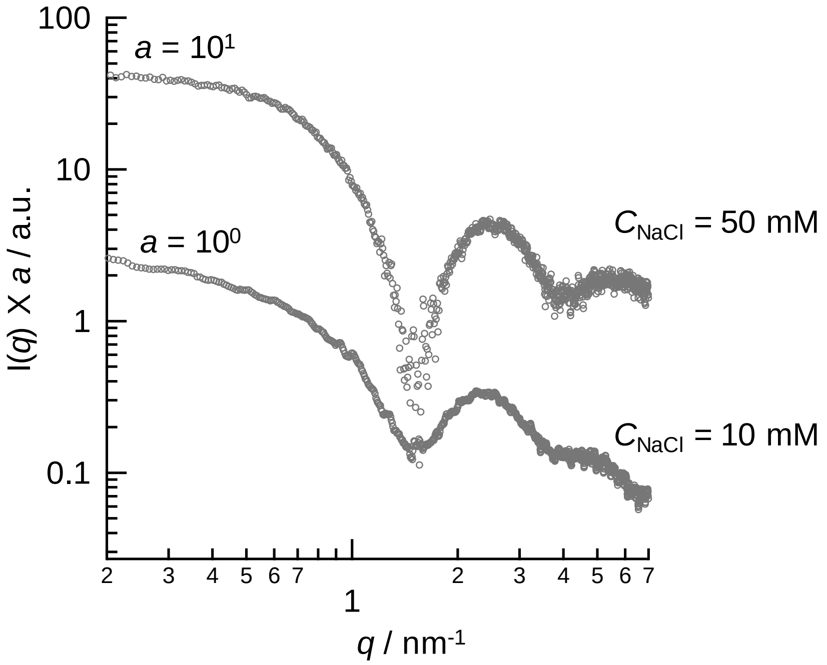

Figure S9-2. SAXS profiles of sufactin micelles in 10 and 50 mM NaCl aqueous solutions.

(S10) Optimal configuration of N points on a sphere

Tammes problem, i.e., the best packing on a sphere. How can N points $=\{\boldsymbol{x}_1, \boldsymbol{x}_2, \cdots \boldsymbol{x}_N\}$ be distributed on a unit sphere so that they maximize the minimum distance (a_N) between any pair of points? Mathematically, this is a minimax theorem of spherical distance called the Tammes problem:

$$a_N := \max_{X_N} \min_{i<j} |\boldsymbol{x}_i - \boldsymbol{x}_j| \qquad (\mathrm{S10-1})$$

The maximum distance a_N is called the covering radius. Obviously, for $N = 2$, $a_N = \pi$ when two points are located at each pole of the sphere. For $N = 3, 4, 6,$ and 12, it can be shown that:

$$a_N = \cos^{-1}\frac{\cos\alpha_N}{1-\cos\alpha_N} \quad \text{where } \alpha_N = \frac{N}{N-2}\frac{\pi}{3} \qquad (\mathrm{S10-2})$$

The vertices of the regular tetrahedron, octahedron, and icosahedron of Platonic solids are identical to the optimized configurations for $N = 4$, 6, and 12. For $N = 8$, the configuration is not a hexahedron, but an augmented triangular prism (*i.e.*, a skewed cube). For the other N-values, exact or numerical results are available. It can be shown that a_N corresponds to the radius of the spherical cap that has the closest packing. The fraction of the spherical surface covered by N identical spherical caps, *i.e.,* the coverage density, (D), is given by:

$$D(N) = \frac{N}{2}\left(1 - \cos\frac{a_N}{2}\right) \qquad (\mathrm{S10-3})$$

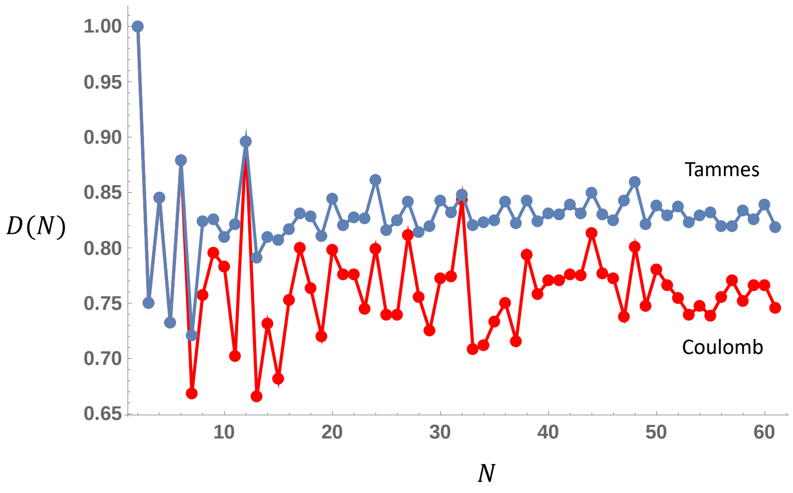

Figure S10-1. $D(N)$ For N ranging from 1 to 62. Note that $D(N)$ is always less than the value at $D(12)$. Certain numbers produce a local maximum and are identical to the number of the vertices in Platonic, Archimedean, and other solids as shown below.

4	tetrahedron	12	icosahedron
6	octahedron	24	snub cube
8	square antiprism	32	soccer ball

Thomson problem. N charged points, $\boldsymbol{X}_N$, are placed on the surface of a sphere in a way that minimizes the total (dimensionless) Coulomb energy of the system, $E(\boldsymbol{X}_N)$:

$$E(\boldsymbol{X}_N) = \sum_{i=1}^{N-1} \sum_{j=i+1}^{N} \frac{1}{|\boldsymbol{x}_i - \boldsymbol{x}_j|} \qquad (S10-4)$$

This is the Thomson problem. For certain values of N, exact numerical coordinates are available. $N = 3,\ 4, 5, 6, 12, 24,$ and 32 give the same results as the Tammes problem. When the interaction potential is expressed by $\emptyset(r) = r^{-\alpha}$, the Thomson and Tammes problems correspond to $\alpha = 1$ and $\alpha = \infty$, respectively. Therefore, the two problems are closely related. The coverage density determined from $E(\boldsymbol{X}_N)$ is plotted in Figure S10-1. Figure S10-2 plots the minimized $E(\boldsymbol{X}_N)$ against N, which increases monotonically with increasing N.

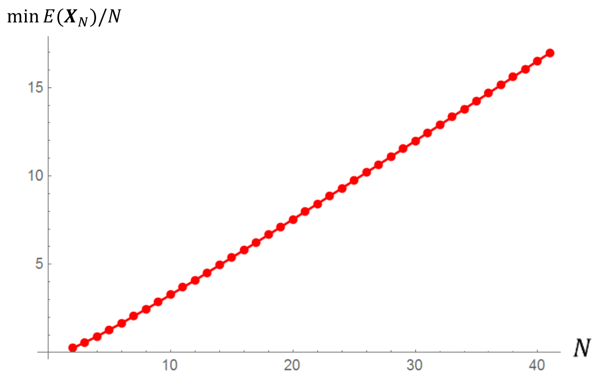

Figure S10-2. Dependence of the Coulomb energy on the number of points.

References

(S10-1) Erber, T.; Hockney, G. M., Complex Systems: Equilibrium Configurations of N Equal Charges on a Sphere (2 ≤ N ≥ 112). In Advances in Chemical Physics, John Wiley & Sons, Inc.: 2007; pp 495-594.
http://www.mathsisfun.com/geometry/polyhedron-models.html
https://en.wikipedia.org/wiki/Icosahedral_symmetry
http://www.mathpages.com/home/kmath005/kmath005.htm
https://www.maths.unsw.edu.au/about/distributing-points-sphere

(S11) Thermodynamics of spherical micelles

Tanford model: For spherical micelles, the standard free energy change is comprised of three major terms as shown in Eq. S11-1, which corresponds to a situation where a surfactant molecule is transferred from an infinitely dilute (*i.e.*, molecularly dispersed) state in water to an aggregate with a finite value of N (ref S12-1-3):

$$\left(\frac{\Delta\mu_N^\circ}{kT}\right) = \left(\frac{\Delta\mu_N^\circ}{kT}\right)_{\text{trasfer}} + \left(\frac{\Delta\mu_N^\circ}{kT}\right)_{\text{interface}} + \left(\frac{\Delta\mu_N^\circ}{kT}\right)_{\text{head-repulsion}} \qquad (S11-1)$$

The first term accounts for the favorable transfer of the tail from water to the hydrophobic atmosphere of the aggregate core. Therefore, it has a negative contribution and depends only on the degree of hydrophobicity of the tail; it is considered to be constant for a given system. The second term is related to the fact that there is residual contact with water at the surface. Assuming that the contact free energy per unit area is constant, this term is an increasing function of the interfacial micelle area, $4\pi R^2/N$, at fixed N, where R is the radius of the core. This is true, because the contact probability between water and the tails increases with increasing interfacial area. The third term introduces a positive contribution representing the repulsive interactions between head groups due to a combination of steric interactions, electrostatic interactions, and ion–ion repulsions. The repulsion increases when the head groups are close to one another. Therefore, the third term is a decreasing function of the distance between adjacent head groups. Although more detailed free energy models have been formulated and have predicted experimental results more accurately, Eq S11-1 is the basic framework provided by Tanford. Within the framework of the packing parameter concept, the aggregation number can be expressed by (see the main text):

$$N_{agg} = \frac{4\pi h^2}{a_e} \qquad (S11-2)$$

Therefore, $4\pi R^2/N_{agg} = a_e$, when $R = h$. Here, a_e is the equilibrium area per molecule at the aggregate interface. Tanford proposed an expression with an inverse dependence on a_e for the third term. Therefore, Eq (S11-1) can be rewritten as:

$$\left(\frac{\Delta\mu_N^\circ}{kT}\right) = \left(\frac{\Delta\mu_N^\circ}{kT}\right)_{\text{trasfer}} + \left(\frac{\sigma}{kT}\right)a_e + \left(\frac{\alpha}{kT}\right)\frac{1}{a_e} \qquad (S11-3)$$

Here *assumed* and the headgroup repulsion parameter, respectively. We can obtain the equilibrium value of a_e by use of the condition:

$$\frac{\partial}{\partial a_e}\left(\frac{\Delta\mu_{N_{agg}}^\circ}{kT}\right) = 0 \qquad (S11-4)$$

which gives $a_e = \sqrt{\alpha/\sigma}$. When $\Delta\mu_N^\circ$ has its minimum value at $N = N_{agg}$, the variation of $\Delta\mu_N^\circ$ may be written in parabolic form as $\Delta\mu_N^\circ - \Delta\mu_{N_{agg}}^\circ \sim (N - N_{agg})^2$. Therefore, the standard deviation in the aggregation number (SD) is given by

$$SD \sim \sqrt{N_{agg}} \qquad (S11-5)$$

<u>**Considerations from the Tammes best packing problem.**</u> In the conventional model, the interfacial free energy term is proportional to a_e. This is based on the assumption that the hydrophilic head groups may not prevent unfavorable contact between water and tails and that the contact probability will increase with increasing interfacial area. However, in terms of the Tammes best packing problem (S10), the contact probability is determined by the coverage efficiency produced by a certain number of circles. This can be expressed by the coverage density, $D(N)$. Therefore, the second tem in Eq S11-1 will equal:

$$\left(\frac{\Delta\mu_N^\circ}{kT}\right)_{\text{interface}} = \left(\frac{\sigma}{kT}\right)\frac{4\pi R^2}{N_{agg}}[1 - D(N)] \qquad (S11-6)$$

Although we accept the relation $4\pi R^2/N_{agg} = a_e$, the $1 - D(N)$ term produces the very complicated phenomenon shown below. Therefore, we presume that it is impossible to obtain a_e by use of Eq S11-4.

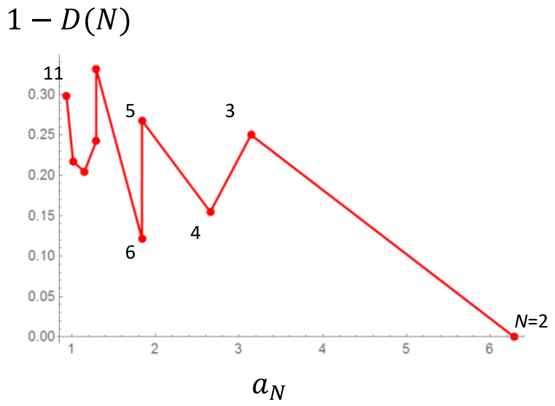

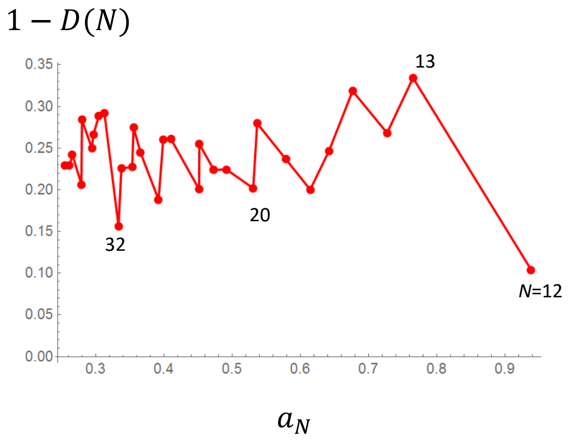

The above discussion is based on the assumption that the interface between the hydrophobic and hydrophilic domains of surfactants can be regarded as a spherical cap with hard-core potentials, the shape of the cap does not change and all caps have the same radius. Real surfactants may take various conformations and thus may deform to avoid unfavorable contact between waters and tails. However, our observation of the discreteness in N_{agg} and the relevance to the Tammes problem suggest that the contact free energy per unit area (σ) cannot be considered constant at low N_{agg}, which thus should be taken into account. Also, the hard-core potential is not a necessary condition for an oscillating $D(N)$ pattern. A similar simulation with the Coulomb potential exhibits local maxima of $D(N)$ at $N = 4, 12, 16,$ and 20. While one might assume that at large N_{agg} σ is constant. It is not clear at the moment how large N_{agg} must be to consider σ to be constant; this may depend on the chemical structure. We would like to point out that there are maxima of $D(N)$ at $N = 32$ and 48 and these numbers are normally observed N_{agg} values for conventional surfactants such as alkyl sulfates {Jusufi, 2012}, suggesting that σ may not be constant, but depend on N even in these systems.

References

(S11-1) Tanford, C., The Hydrophobic Effect: Formation of Micelles and Biological Membranes, 2nd ed. Wiley-Interscience: New York, 1980.
(S11-2) Israelachvili, J. N., Intermolecular and surface forces. Academic press London: 1992; Vol. 450.
(S11-3) Nagarajan, R.; Ruckenstein, E., Theory of surfactant self-assembly: a predictive molecular thermodynamic approach. Langmuir 1991, 7 (12), 2934-2969.
(S11-4)
Erber, T.; Hockney, G. M., Complex Systems: Equilibrium Configurations of N Equal Charges on a Sphere ($2 \leq N \geq 112$). In Advances in Chemical Physics, John Wiley & Sons, Inc.: 2007; pp 495-594.
(S11-5) Jusufi, A., LeBard, D. N., Levine, B. G. & Klein, M. L. Surfactant concentration effects on micellar properties. *J Phys Chem B* 116, 987-991, doi:10.1021/jp2102989 (2012).

(S12) Computer chemistry

In our previous paper {Fujii, 2012 }, the scattering pattern of the micelle of PACaL3 at pH 1.2 up to a resolution of $q \cong 5$ nm^{-1} was used to produce dummy atom models with the program DAMMIN, an *ab initio* shape reconstruction program for solution scattering.{Svergun, 1999 #1107} A MOPAC optimized atomic model of PACaL3 was used for the construction of a rigid body model of the hexameric micelle with the program SASREF {Petoukhov, 2006 #2953} (S12) which shows good agreement with the experimental SAXS data. Using this rigid body models as a guide, we designed symmetric models (with PACaL3 monomers arranged on the faces of a cube), that were used as starting models for a series of molecular dynamics (MD) simulations. Figure 6a plots the total energy (E_{tot}) of the system and the Jaccard index ($J_{\mathrm{ind.}}$) between the experimental SAXS profile and the one calculated from each MD model with CRYSOL {Svergun, 1995 #2960} as a function of the elapsed time of MD. As expected, MD shows that the PACaL3 hexameric micelle is actually constantly changing its structure, including rapid inter-conversion between the *C4v* and *C2v* configuration of the calix[4]arene ring{Fujii, 2012 #1390}. While E_{tot} oscillates around a constant value, $J_{\mathrm{ind.}}$ fluctuates considerably. The closer the $J_{\mathrm{ind.}}$ value is to 1, the better the agreement between the experimental and the calculated SAXS profiles. Figure 6b compares three profiles with $J_{\mathrm{ind.}}$ = 0.9973, 09967, and 0.975 with the experimental scattering pattern, showing that the profile with $J_{\mathrm{ind.}}$ = 0.997 is in much better agreement with the data. Because SAXS patterns are an averaging of all the different conformations the micelle adopts, one would expect that an ensemble of all the MD conformations would give a better fit to the experimental data. However, this is not what we observed (S12). This discrepancy may indicate that the MD simulations overestimate the thermal fluctuations of the atomic positions.

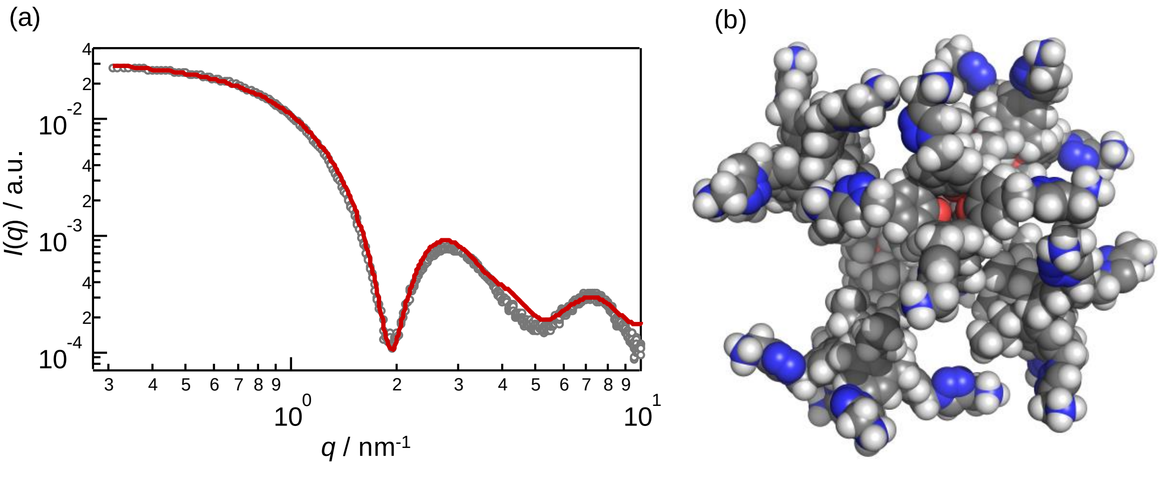

Figure S12-1. (a) Comparison of the SAXS experimental data (gray circles) of PACCaL3 at pH 1.2 and the calculated profiles from a rigid body model (b).

Rigid body and MD simulations

Rigid body model calculations of micelles were performed by the program SASREF with the original conformation of the molecules calculated with MOPAC. Six molecules of PACaL3 were used for the rigid body model, and distance restraints between molecules were introduced to insure that the tail groups point to the inside of the structure.

All MD simulations were carried out using the Amber 11 program suite on a CUDA workstation with the parameters of the General Amber Force Field (GAFF). The initial geometry of the molecule was optimized at the HF/6-311G(d,p) level with the help of Gaussian 09. The partial charges of atoms were determined with the RESP ESP charge Derive (R.E.D.) tools III.4. Six PACaL3 molecules were initially arranged in cubic symmetry with each molecule occupying one face of a cube. The initial arrangement was as tight as possible without steric clashes between molecules. The molecules were immersed in a TIP3P box with periodic boundary conditions, and all simulations were performed in explicit solvent at 300 K and a constant pressure of 1 atm after initial minimization and stepwise heating at constant volume. The possibility of implicit solvent simulations was explored, but the instability of the micelles in this environment made it an unviable option (the molecules dissociate shortly after starting the simulations). Simulations were performed up to 60 ns with time steps of 1 or 2 fs.

The SAXS patterns of structures from snapshots taken at regular intervals during the simulations were computed with CRYSOL. While it was possible to use the water molecules closest to the micelles as the hydration shell, we opted for CRYSOL's built-in facility to account for scattering of the hydration shell. It has the advantage of being computationally much cheaper, and, because of the difficulty and uncertainty in determining which individual water molecules belong to the hydration shell, it leads to somewhat better results in terms of the fit to experimental data.

Reference

EMBL, Biological small angle scattering
http://www.embl-hamburg.de/biosaxs/software.html

(S13) The Jaccard similarity coefficient

The Jaccard index, also known as the Jaccard similarity coefficient, is a statistic used for comparing the similarity and diversity of sample sets. The Jaccard coefficient measures similarity between finite sample sets:

$$J_{ind} = \frac{\sum_{i=0}^{N}[\log I(q_i) \times \log I_c(q_i)]}{\sum_{i=0}^{N}[\log I(q_i)]^2 + \sum_{i=0}^{N}[\log I_c(q_i)]^2 - \sum_{i=0}^{N}[\log I(q_i) \times \log I_c(q_i)]}$$

Here, $I(q_i)$ and $I_c(q_i)$ are the observed and calculated scattering intensities at the same q_i.

The commonly used statistic is a chi-squared test, which is expressed in our case as:

$$\chi^2 = \sum_{i=0}^{N} \frac{[\log I(q_i) - \log I_c(q_i)]^2}{\log I(q_i)}$$

In our case, the chi-squared test gives the same result as the Jaccard.

(S14) ¹H NMR spectra of calix[4]arene-based lipids

¹H NMR spectrum of QACaL3

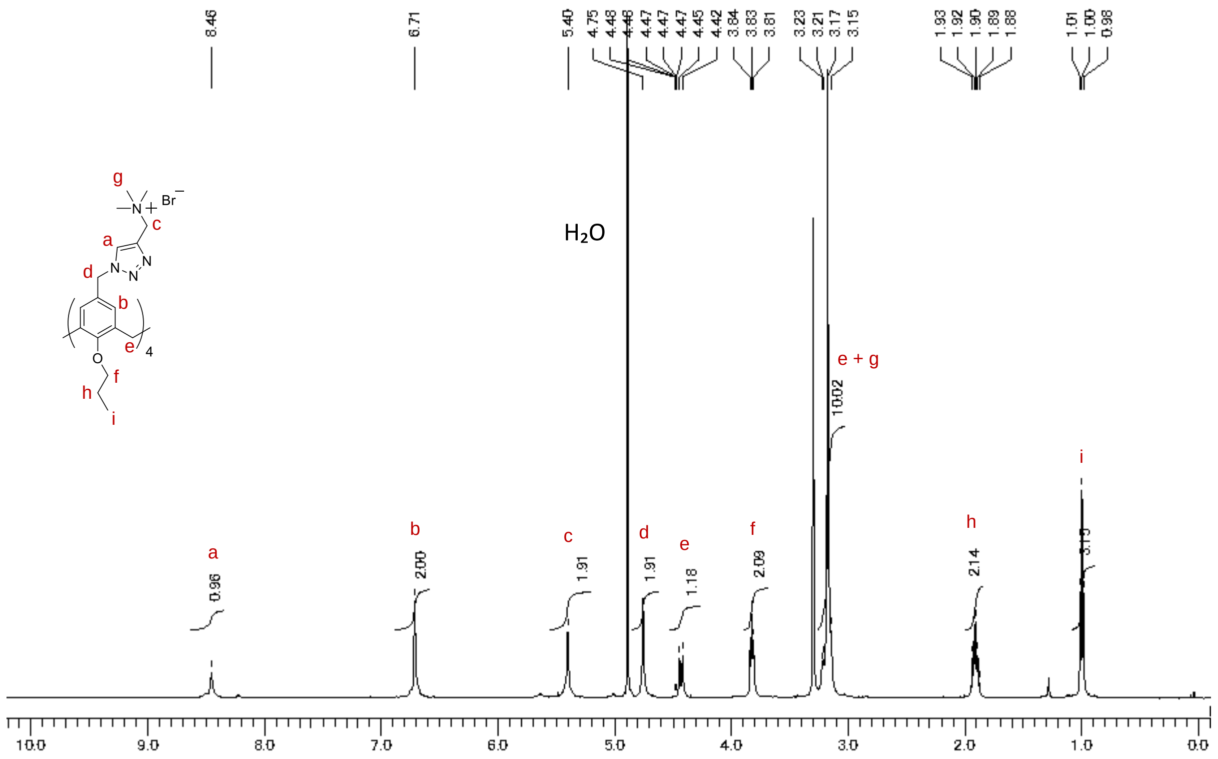

¹H NMR spectrum of QACaL4

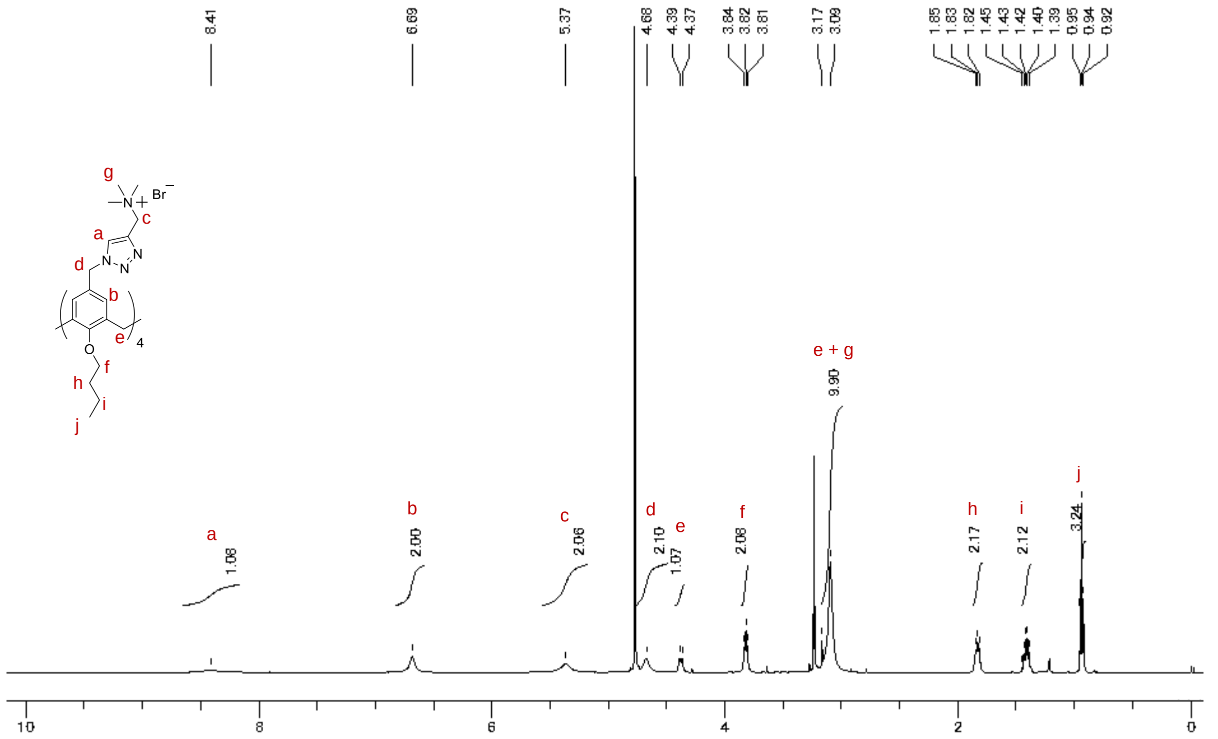

^{1}H NMR spectrum of QACaL5

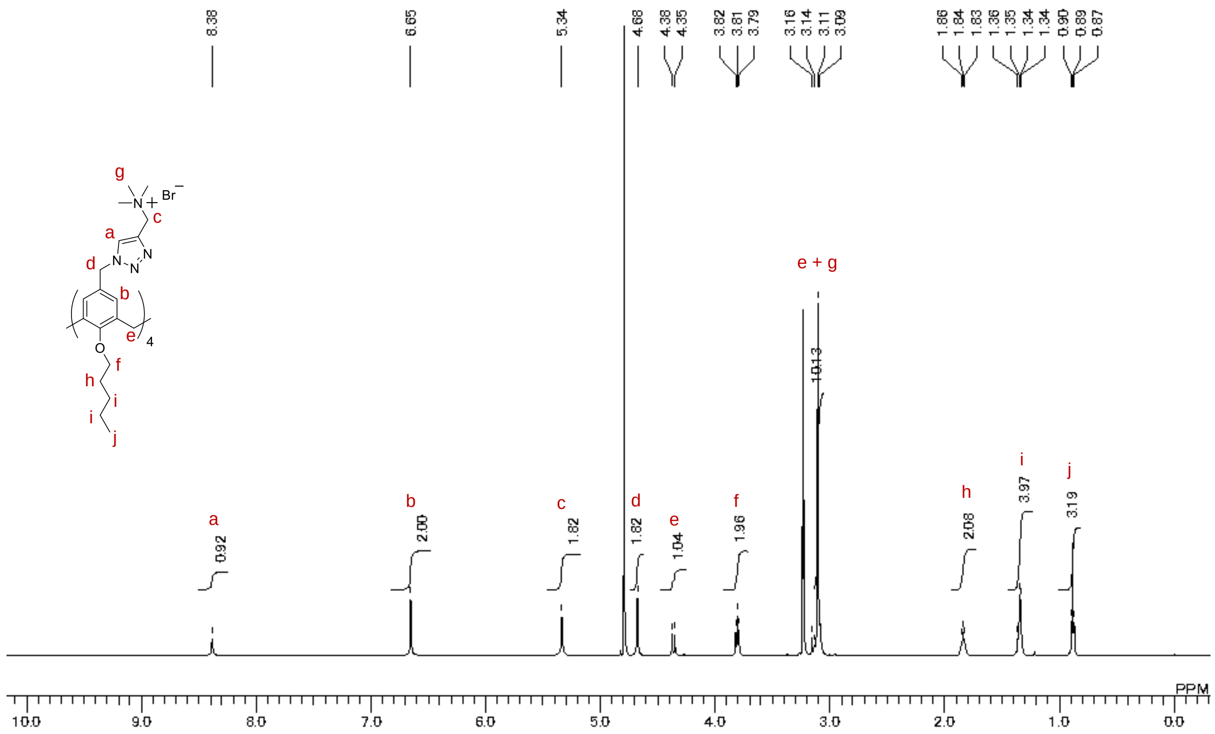

^{1}H NMR spectrum of QACaL6

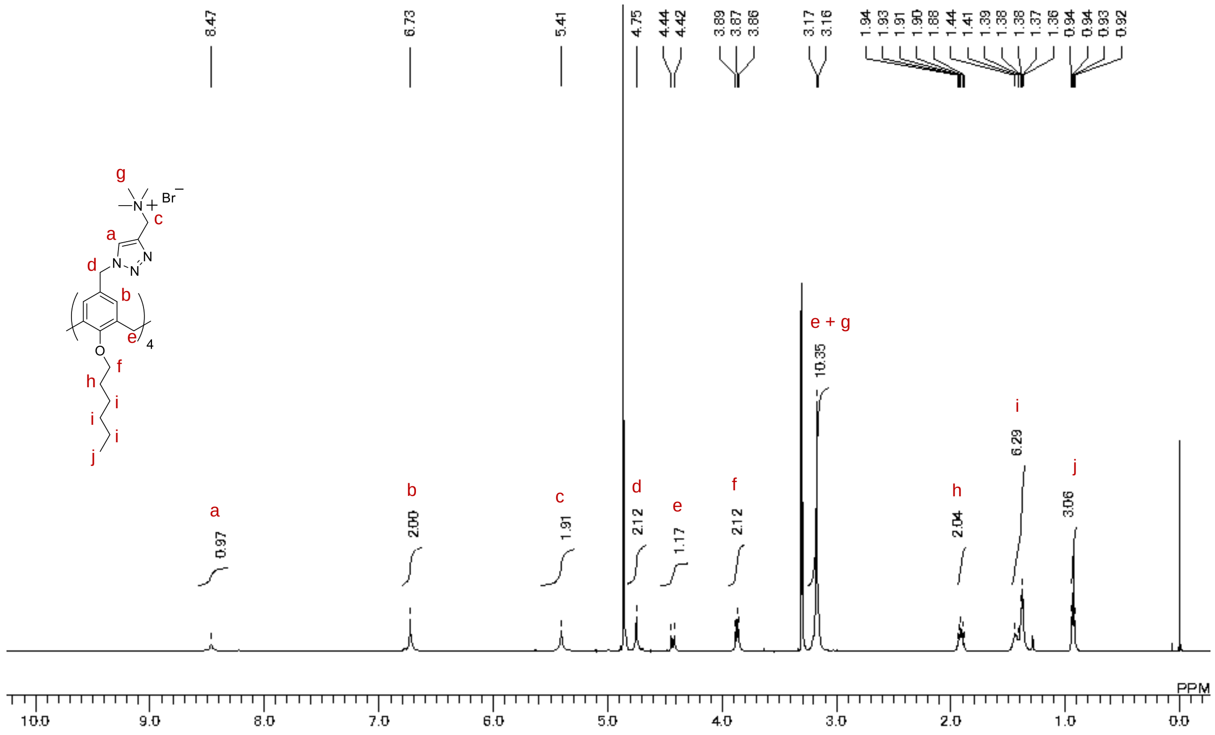

^{1}H NMR spectrum of QACaL7

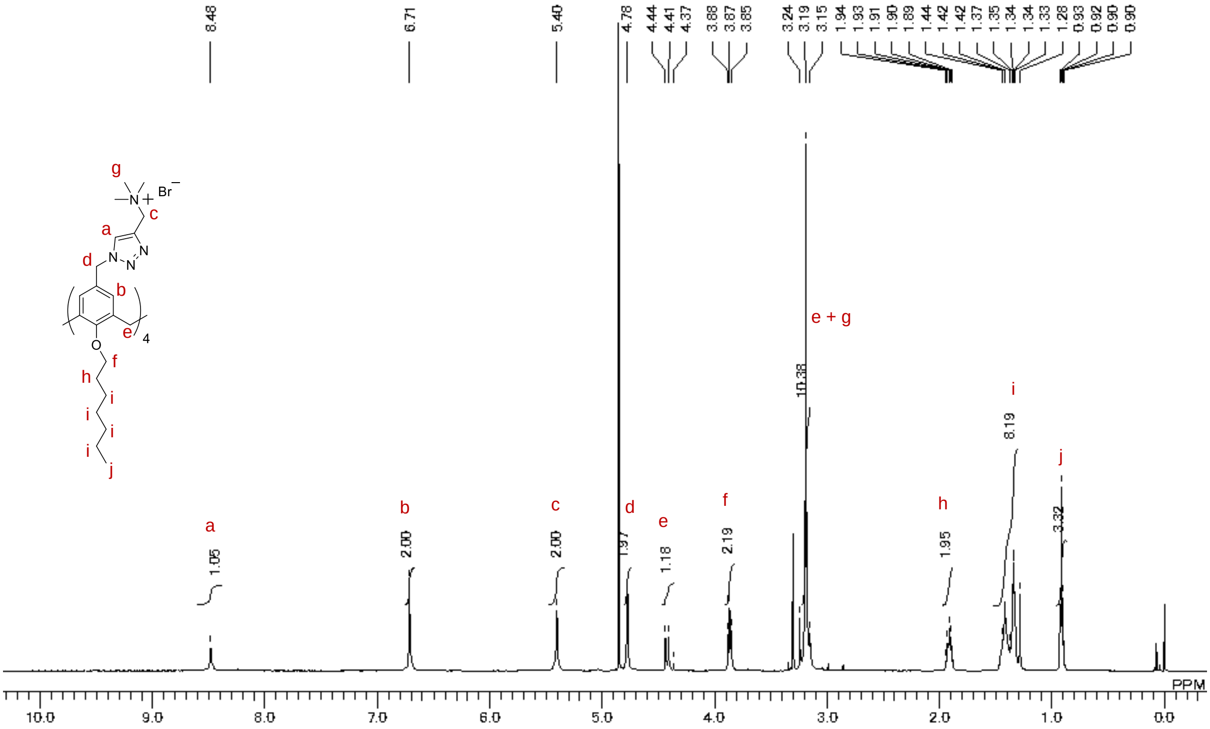

^{1}H NMR spectrum of ECaL3

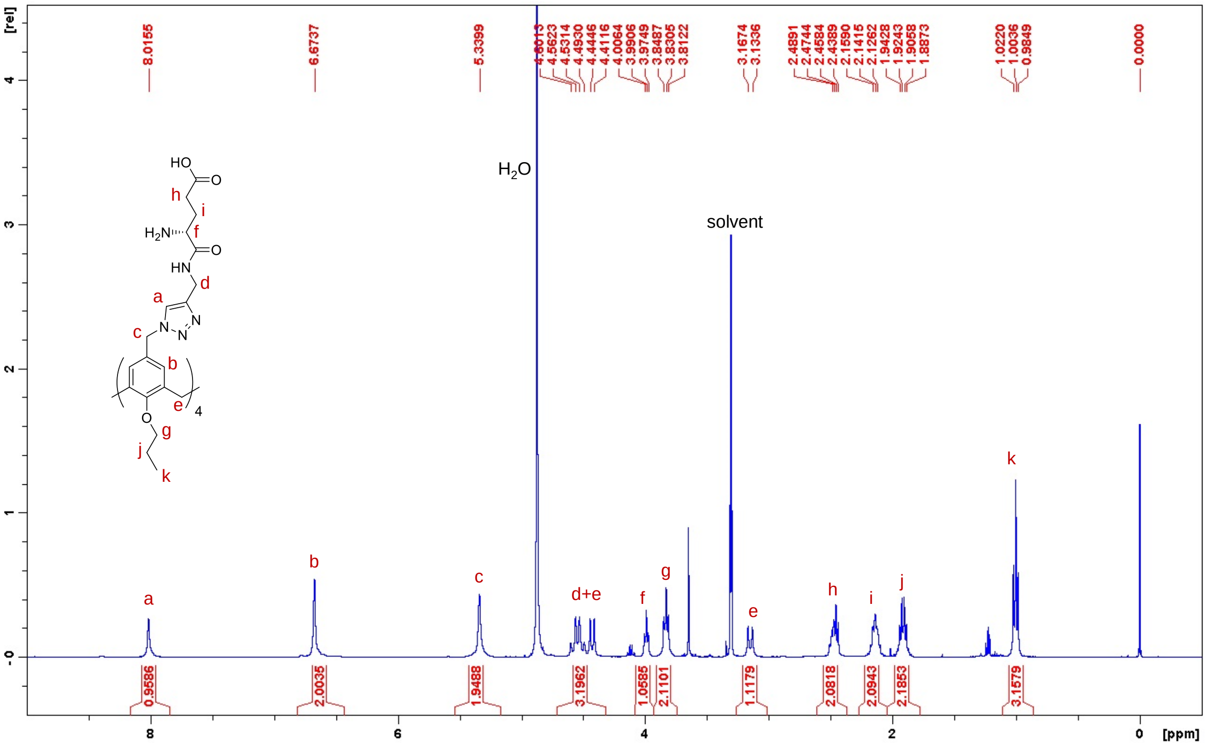

^{1}H NMR spectrum of G1CaL3

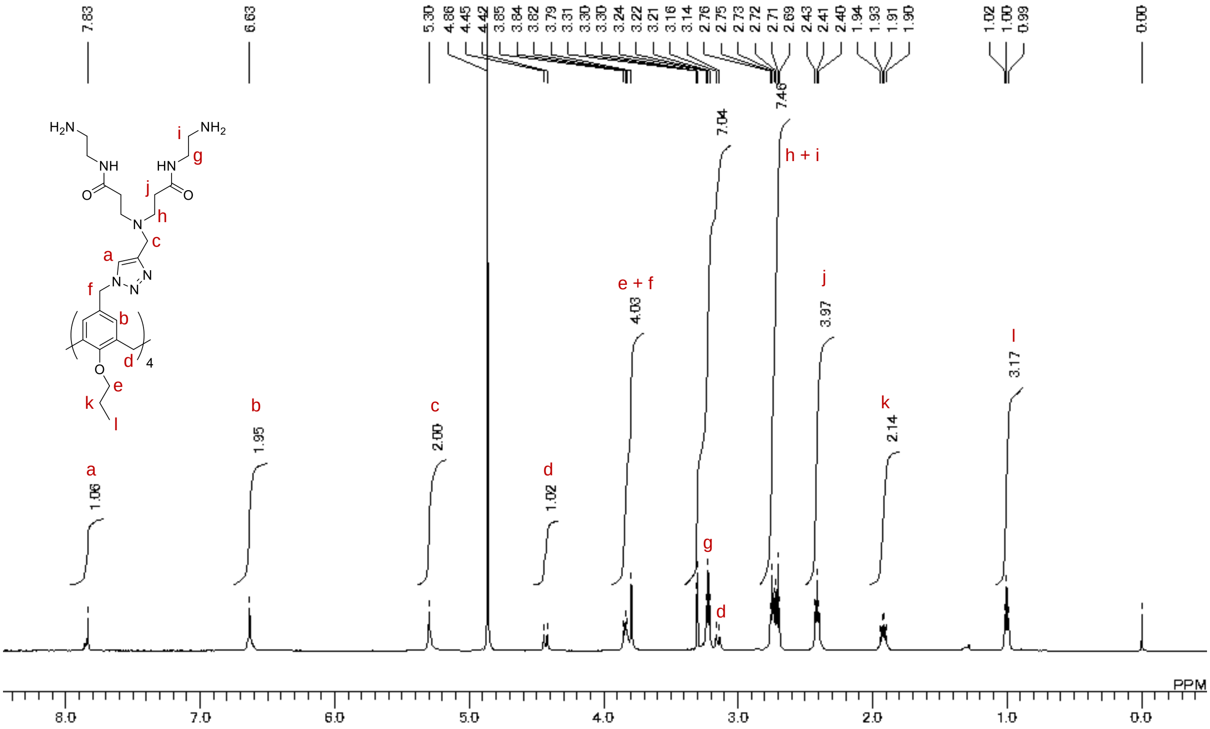

^{1}H NMR spectrum of PEG_{550}CaL5

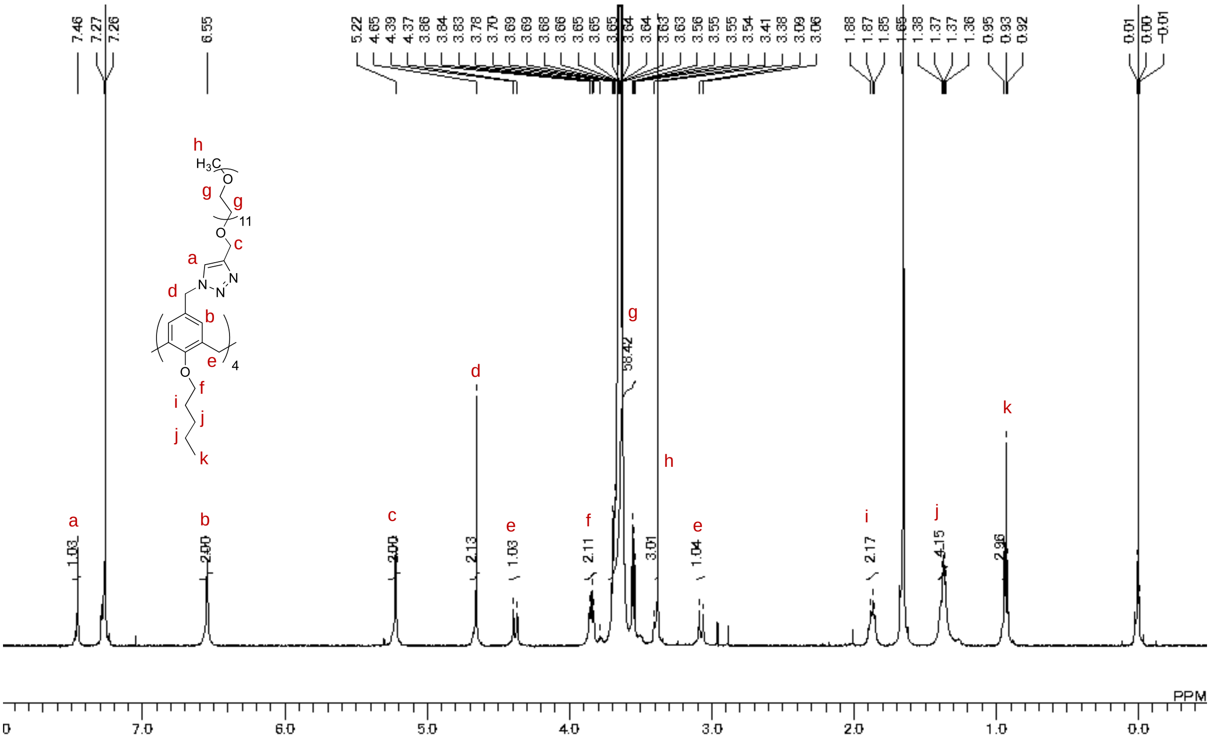

^{1}H NMR spectrum of $PEG_{1k}CaL5$

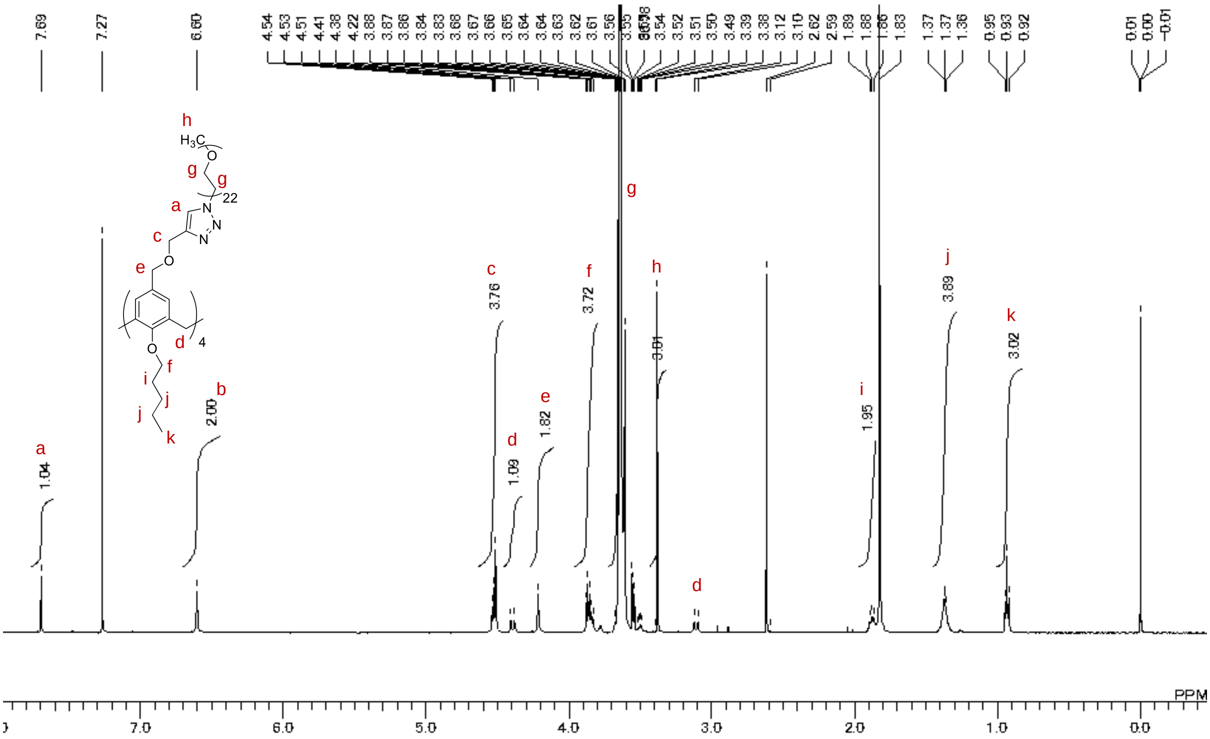

^{1}H NMR spectrum of $PEG_{2k}CaL5$

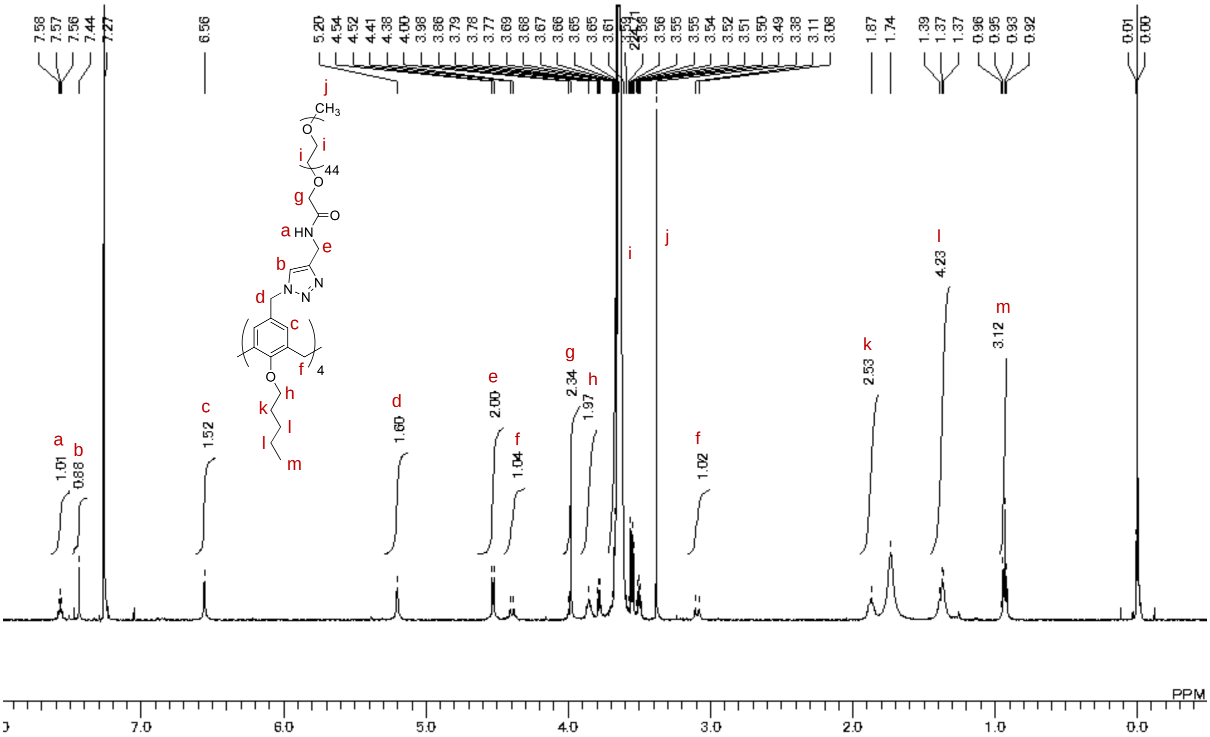

Supplement: Supplementary Information [file srep44494-s1.pdf]
